# Temperature Shift and Host Cell Contact Up-Regulate Sporozoite Expression of *Plasmodium falciparum* Genes Involved in Hepatocyte Infection

**DOI:** 10.1371/journal.ppat.1000121

**Published:** 2008-08-08

**Authors:** Anthony Siau, Olivier Silvie, Jean-François Franetich, Samir Yalaoui, Carine Marinach, Laurent Hannoun, Geert-Jaan van Gemert, Adrian J. F. Luty, Emmanuel Bischoff, Peter H. David, Georges Snounou, Catherine Vaquero, Patrick Froissard, Dominique Mazier

**Affiliations:** 1 INSERM, U511, Paris, France; 2 Université Pierre et Marie Curie-Paris 6, UMR S511 Paris, France; 3 Service de Chirurgie Digestive, Hépato-Bilio-Pancréatique et Transplantation Hépatique, Hôpital Pitié-Salpêtrière, Paris, France; 4 Department of Medical Microbiology, University Medical Centre, Nijmegen, The Netherlands; 5 PF2-Genopole, Institut Pasteur, Paris France; 6 Unité d'Immunologie Moléculaire des Parasites, CNRS URA 2581, Département de Parasitologie, Institut Pasteur, Paris, France; 7 Laboratoire de Parasitologie Comparée et Modèles Expérimentaux, Muséum National d'Histoire Naturelle, Paris, France; 8 AP-HP, Groupe hospitalier Pitié-Salpêtrière, Service Parasitologie-Mycologie, Paris, France; Cornell University, United States of America

## Abstract

*Plasmodium* sporozoites are deposited in the skin by *Anopheles* mosquitoes. They then find their way to the liver, where they specifically invade hepatocytes in which they develop to yield merozoites infective to red blood cells. Relatively little is known of the molecular interactions during these initial obligatory phases of the infection. Recent data suggested that many of the inoculated sporozoites invade hepatocytes an hour or more after the infective bite. We hypothesised that this pre-invasive period in the mammalian host prepares sporozoites for successful hepatocyte infection. Therefore, the genes whose expression becomes modified prior to hepatocyte invasion would be those likely to code for proteins implicated in the subsequent events of invasion and development. We have used *P. falciparum* sporozoites and their natural host cells, primary human hepatocytes, in *in vitro* co-culture system as a model for the pre-invasive period. We first established that under co-culture conditions, sporozoites maintain infectivity for an hour or more, in contrast to a drastic loss in infectivity when hepatocytes were not included. Thus, a differential transcriptome of salivary gland sporozoites versus sporozoites co-cultured with hepatocytes was established using a pan-genomic *P. falciparum* microarray. The expression of 532 genes was found to have been up-regulated following co-culture. A fifth of these genes had no orthologues in the genomes of *Plasmodium* species used in rodent models of malaria. Quantitative RT-PCR analysis of a selection of 21 genes confirmed the reliability of the microarray data. Time-course analysis further indicated two patterns of up-regulation following sporozoite co-culture, one transient and the other sustained, suggesting roles in hepatocyte invasion and liver stage development, respectively. This was supported by functional studies of four hitherto uncharacterized proteins of which two were shown to be sporozoite surface proteins involved in hepatocyte invasion, while the other two were predominantly expressed during hepatic parasite development. The genome-wide up-regulation of expression observed supports the hypothesis that the shift from the mosquito to the mammalian host contributes to activate quiescent salivary gland sporozoites into a state of readiness for the hepatic stages. Functional studies on four of the up-regulated genes validated our approach as one means to determine the repertoire of proteins implicated during the early events of the *Plasmodium* infection, and in this case that of *P. falciparum*, the species responsible for the severest forms of malaria.

## Author Summary

Sporozoites, the infective form of the malaria parasites *Plasmodium*, are deposited in the skin by *Anopheles* mosquitoes. They then find their way to the liver where they specifically invade hepatocytes, in which they develop to yield another form, the merozoite, infective to red blood cells. Relatively little is known of the molecular interactions during these initial obligatory phases of the infection. We studied the changes in gene expression in sporozoites, from the parasite species *P. falciparum* that infects humans, in an *in vitro* system where they were co-cultured with their natural host cells, primary human hepatocytes. The whole genome transcriptome profiling carried out led to the identification of 532 genes that were up-regulated following co-culture. This genome-wide up-regulation of expression supports the hypothesis that the shift from the mosquito to the mammalian host contributes to activate quiescent salivary gland sporozoites into a state of readiness for the hepatic stages. Functional studies on four of the up-regulated genes we identified validated our approach as one means to determine the repertoire of proteins implicated during the early events in the infection by *P. falciparum*, the species responsible for the severest forms of malaria.

## Introduction

Protozoan parasites of the genus *Plasmodium* are the causative agents of malaria, the most devastating parasitic disease in humans. The infection is initiated when *Plasmodium* sporozoites are deposited in the skin of their vertebrate hosts through the bite of an infected female *Anopheles* mosquito. The sporozoites released from oocysts in the mosquito migrate to the salivary gland where they lodge in the acinar lumen ready for inoculation during a feeding bite. In the mosquito, salivary gland sporozoites retain infectivity for many days, and even weeks under optimal conditions of temperature and humidity. Once inoculated into the mammalian host, the sporozoites migrate to the liver where they cross the sinusoid wall and subsequently migrate through several hepatocytes before infecting a final hepatocyte [1],[2]. Although some sporozoites can reach the blood circulation very rapidly [3], recent studies suggest that the majority trickle out of the injection site over several hours [4]–[6]. There are clear indications that sporozoites retain infectivity at least one hour *in vivo* [3],[6], which contrasts with the rapid loss of infectivity observed when sporozoites are maintained *in vitro* at 37°C [7]. Incubation at the higher temperature does not necessarily lead to the death of all sporozoites, since it was shown that about one in 10 sporozoites incubated for 24 hours at 37°C in the presence of serum transform into forms morphologically indistinguishable from early exo-erythrocytic parasites [8]. These observations, indicate that when shifted from the insect to the mammalian host environment, the relatively quiescent salivary gland sporozoites are somewhat activated in preparation for hepatocyte infection and that their infectivity is preserved until then.

The above observations led us to hypothesise that both the shift in temperature and contact with host cells contribute to the preservation of sporozoite infectivity and its activation. In that case, the genes whose expression is modified by the shift to the mammalian host environment could be identified by whole genome transcriptome analyses. Sufficient quantities of parasite material necessary for such analyses cannot be obtained from *in vivo* infections. Therefore, we have subjected sporozoites to host-like conditions *in vitro*. We have opted for the hepatocyte to represent host cell contact, as hepatocytes are the only cell type in which the sporozoite develops to maturity in the host. Furthermore, it has been recently shown that specific molecular interactions with hepatocyte activate sporozoites for invasion [9]. Although *Plasmodium* species that infect rodents offer an adequate and practical model for the study of pre-erythrocytic stages, about a third of the genes found in *P. falciparum*, the parasite associated with the bulk of mortality and morbidity due to malaria, do not have orthologues in these species [10]. Therefore, the studies presented here were conducted using *P. falciparum* sporozoites and human primary hepatocytes.

## Results

### Combined temperature shift and host cell contact preserve sporozoite infectivity

In order to ensure that data whole genome transcription analyses were valid biologically, we considered it necessary to ensure that the sporozoites experimentally subjected to host-like conditions were still infectious. For *P. falciparum* sporozoites biological investigations are ethically restricted to *in vitro* infections thus, infectivity assays can only be conducted in primary human hepatocytes [11].

**Figure 1:**
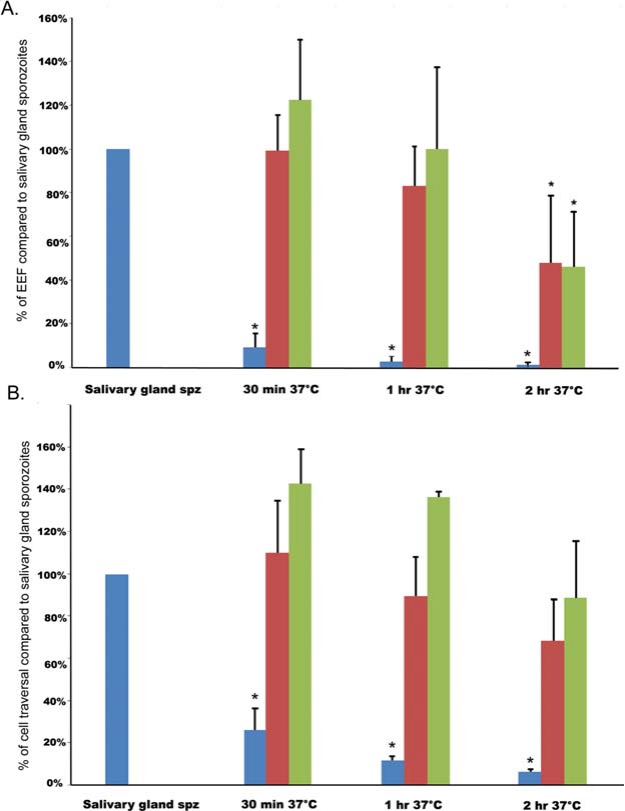
Infection and cell traversal abilities of sporozoite incubated at 37°C in the absence or the presence of hepatocyte or skin cell. A. Infectivity of *P. falciparum* sporozoites pre-incubated for 30 minutes, 1 or 2 hours at 37°C in physiological medium in the absence (blue), or in the presence of human primary hepatocytes (red), or human skin keratinocytes (green). Infectivity is expressed as a percentage of the values obtained with control salivary gland sporozoites kept at 4°C for 2 hours before running the infectivity assay (546±289 hepatic schizonts per well were obtained for these control sporozoites). B. Cell traversal ability of pre-incubated sporozoites (as above) was determined by enumeration of cells wounded by sporozoite passage. Results are expressed as the percentage of the values observed for control salivary gland sporozoites. For the two experiments above, data is expressed as the mean of the values obtained from at least 3 independent assays, each performed in triplicate wells (errors bars denote+/−S.D.). * P<0.05 when compared to salivary gland sporozoites.

In a first series of experiments (Figure 1A), sporozoites were incubated at 37°C in medium supplemented with serum for different periods of time, and then tested in the standard sporozoite infectivity assay (see Materials and Methods). Infectivity was substantially reduced by 30 minutes of incubation (15% of control) and nearly fully abrogated after two hours (2% of control). When incubation at 37°C was conducted in the presence of a cell line (HaCaT) derived from human skin keratinocytes or of primary human hepatocytes, the loss of infectivity could be fully prevented during the first hour of incubation (37°C), and >50% of the sporozoites retained infectivity by the end of the second hour of incubation (Figure 1A). In parallel, we analysed the ability of the sporozoites to migrate through cells, a phenomenon associated with successful infection [12]. Incubation of sporozoites at 37°C in the absence of host cells was similarly shown to adversely affect sporozoite migration ability (Figure 1B), whereas in the presence of skin keratinocytes or primary hepatocytes *P. falciparum* sporozoite migration ability was mostly preserved after one hour incubation (Figure 1B).

In one final experiment, it was established that when the initial incubation periods of the standard sporozoite infectivity assay were carried out at room temperature (See Materials and Methods), the efficiency with which sporozoites invade human primary hepatocytes was drastically reduced (<25 infected hepatocytes per well, data not shown).

These observations demonstrated that efficient hepatocyte infection requires a shift in temperature (to 37°C), which on its own would lead to a rapid loss of infectivity *in vitro*. However, this loss was substantially delayed when the incubation at 37°C was carried out in the presence of human cells.

### Transcriptome profiling of *P. falciparum* sporozoites co-cultured with human hepatocytes at 37°C

In order to explore the molecular basis for differences between quiescent salivary gland and invasion-ready *P. falciparum* sporozoites, their transcriptomes were sought using a DNA microarray covering the whole *P. falciparum* genome [13]. Given the large numbers of *P. falciparum* sporozoites needed, hepatocytes, rather than keratinocytes, were chosen as the host cells with which the sporozoites were to be incubated. This choice was dictated by two reasons: first, hepatocytes are the specific host cell in which sporozoites develop, and second, primary human hepatocytes directly isolated from the liver were deemed more suitable than a cell line of keratinocytes adapted to *in vitro* cultivation. It was considered likely that the viable and infectious sporozoites obtained by incubation at 37°C in the presence of hepatocytes for 1 hour were physiologically similar to the sporozoites found into the mammalian host immediately prior to productive hepatocyte invasion event.

The transcriptome of *P. falciparum* salivary gland sporozoites had been previously derived using Affymetrix GeneChip arrays [14], though an amplification step was needed to compensate for the relatively small quantities of available RNA. In order to increase signal detection sensitivity without the potential bias inherent to RNA amplification, radiolabeled cDNA [15]–[17] was used to probe an oligonucleotide (70-mer) microarray [13]. In order to optimise specificity, the RNA used was obtained from highly purified salivary gland sporozoites, and an excess of unlabelled RNA purified from uninfected mosquitoes was added to the microarray hybridisation mix.

Whole transcriptome profiling was carried out for *P. falciparum* sporozoites incubated for 1 hour at 37°C with primary human hepatocytes, and for control salivary gland sporozoites. The transcriptome data was derived from three independent experiments each conducted with a different lot of sporozoites. When the microarray datasets for the salivary gland and the incubated sporozoites were compared, the expression levels of 611 genes were found altered by 2-fold or more (Table S1). Steady-state RNA levels were decreased (down-regulated) for 79 genes and increased (up-regulated) for 532 genes. For both the up- and the down-regulated genes, 311 encode hypothetical proteins and 300 encode annotated predicted proteins. These 300 proteins could be classified into 13 families (Table S1), five of which were only represented in the up-regulated subset: proteins associated with parasite invasion (n = 13), metal-ion homeostasis (n = 3), or cytoskeleton (n = 9) and stress responses (n = 15). Members of the remaining eight families were noted in both subsets and included proteins expressed on the surface of the infected RBC surface, or in the parasitophorous vacuolar membrane (PVM) (Table S1). The predicted proteins encoded by 19% of the down-regulated and 23% of the up-regulated genes had been previously detected by mass-spectrometry in *P. falciparum* salivary gland sporozoite extracts [18].

It was considered that the down-regulated genes were less likely to be implicated in downstream events and these were not analysed further at present. The magnitude of the up-regulation observed did not exceed 30-fold, and only 13 genes were found to be up-regulated 10-fold or more. In order to validate the notion that genes with up-regulated expression are indeed likely to be implicated in events leading and contributing to the liver phase of the infection, genes coding for proteins known to play a role in the hepatic stages were sought amongst the up-regulated genes. Several heat shock protein genes were found, including that of HSP-70 found up-regulated in *P. berghei* sporozoites transformed by incubation at 37°C in axenic cultures [8]. Furthermore, the orthologue genes of rodent malaria proteins implicated in liver stage maturation, UIS3 [19] and UIS4 [20], or located in the PVM, EXP-1 [21] and UIS4 [20], were also found. It is interesting to note that four members of the Etramp gene family [22], in addition to that of UIS4, were also present, as was the gene of PfEXP-2 [23] an erythrocytic parasite PVM protein. Several parasite genes that encode proteins known to be involved in hepatocyte invasion, TRAP [24], AMA-1 [25], SPATR [26], SPECT-1 [27], SPECT-2 [28], phospholipase [29] and aldolase [30], were also present within the up-regulated subset.

**Figure 2:**
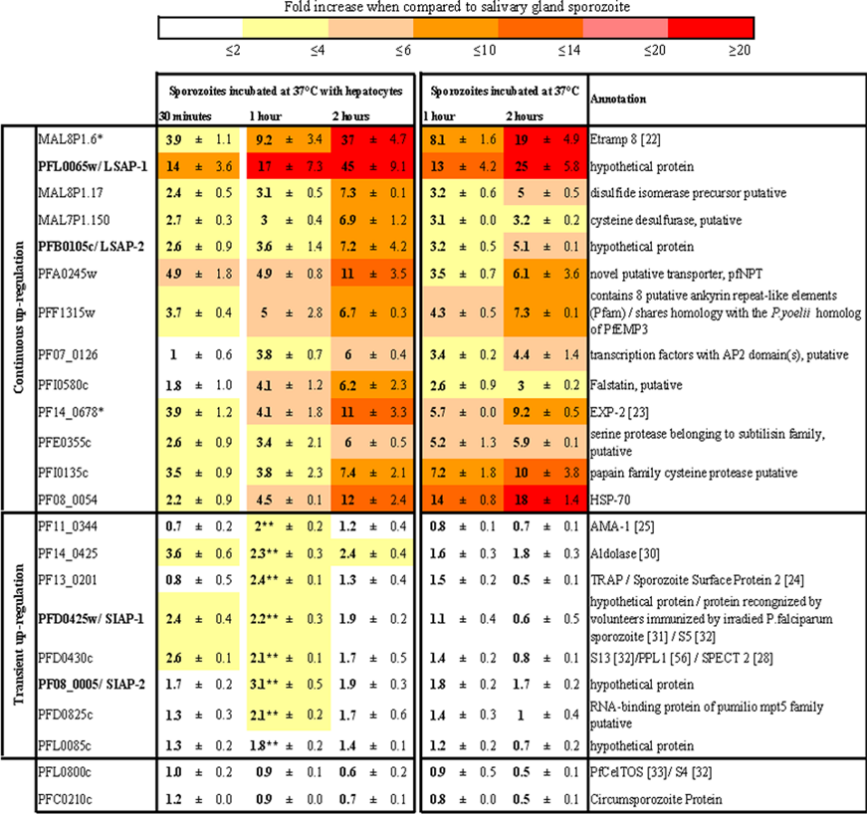
Expression profiling by RT-qPCR of 21 genes up-regulated in activated *P. falciparum* sporozoites. Up-regulation in the expression of 21 genes in sporozoites incubated at 37°C in the presence of absence of primary human hepatocytes for 30 minutes, 1 hour or 2 hours*, was normalized to the expression levels measured for control non-incubated salivary glands sporozoites (values assigned as 1). Up-regulation ratios are represented by a colour gradient. The genes are grouped by the temporal pattern of up-regulation: continuous for those thought to encode proteins involved in parasite metabolism/proteolytic pathway or thought to encode proteins located in the PVM (*), and transient for those encoded proteins implicated in host cell invasion. ** significantly up-regulated (P<0.05) when compared to sporozoites incubated 1hr at 37°C alone. Bold stands for genes characterized using specific antibody.

Despite the difficulties in obtaining simultaneously large numbers of *P. falciparum* sporozoites and primary human hepatocytes, the microarray data was obtained from three replica runs. Only those genes, for which the steady-sate expression level data from the three experiments were similar, were retained as hits. In order to obtain an independent confirmation of the validity of microarray data, 21 up-regulated genes were selected and their expression levels analyzed by Taqman quantitative RT-PCR (RT-qPCR) (Figure 2). Five genes encoded proteins known to be implicated in hepatocyte invasion (TRAP, AMA-1, SPECT-2, and aldolase), or to be expressed in the hepatic stages (HSP-70). Ten other genes were predicted to encode proteins likely to be expressed during hepatic stage development as they are implicated in transcription, transportation, metabolism, proteolytic pathway or are known to be located at the PVM (PfEXP-2 and Etramp 8). The remaining six genes mainly encoded hypothetical proteins, one of which (PFD0425w) was reported to be recognized by immune cells obtained from human immunized by radiation-attenuated sporozoites [31] and is an orthologue of a genes reported to be expressed in *P. yoelii* sporozoites [32]. The proteins encoded by these 21 genes included some that were predicted to have a signal peptide, or a transmembrane domain, both, or neither. Two genes whose expression was found to be unaffected by activation were included as controls: the gene for the circumsporozoite protein (CSP), and PFL0800c that encodes the orthologue of *P. berghei* CelTOS, a micronemal protein involved in cell migration through the sinusoidal cell layer [33]. RT-qPCR analysis preformed with sporozoites incubated for 1 hour at 37°C in the presence of hepatocytes, confirmed the microarray data derived from equivalent material for all the 21 genes tested (Figure 2).

### Temporal patterns of up-regulation and the contribution of hepatocyte contact

The time course of up-regulation for the selected 21 genes was determined by RT-qPCR using RNA purified from salivary gland sporozoites collected after incubation for 30 minutes, one hour, or two hours at 37°C. Incubation was performed either in the presence or in the absence of hepatocytes (Figure 2), because this might provide and indication of the relative contribution of host cell contact and temperature shift to the up-regulation of expression. For sporozoites incubated at 37°C in the presence of hepatocytes, two patterns could be distinguished. A modest transient up-regulation (median up-regulation 1.5, 2.2. and 1.7 for the three time points) was noted for 8 genes, with the peak observed after 30 minutes or after 1 hour incubation, however, no significant up-regulation (<2-fold) was observed for these genes when the sporozoites were solely incubated at 37°C (P<0.05 using the one-way analysis of variance, Figure 2). The four genes encoding proteins known to be implicated in hepatocyte invasion displayed this transient low-level up-regulation pattern. Sustained up-regulation was noted for the other 13 genes, increasing throughout incubation to reach relatively high levels after two hours (median up-regulation 2.7, 4.1 and 7.3 for the three time points). This pattern was equally observed in sporozoites incubated at 37°C either in presence or in the absence of hepatocytes (Figure 2). The group of genes that displayed the continuous up-regulation pattern included all those encoding proteins known or likely to be expressed in infected hepatocytes.

We hypothesised that proteins of unknown function encoded by genes with a transient pattern of up-regulation were likely to be involved in the migratory and invasive processes of the sporozoite in the mammalian host, while those with a continuous up-regulation pattern were likely to be related to parasite development within the hepatocyte.

In order to test this hypothesis, specific antibodies were sought in order to conduct functional analyses. Success in producing recombinant proteins and in raising specific antibodies was met for 4 genes: PFD0425w and PF08_0005 that showed the transient pattern of up-regulation, and PFL0065w and PFB0105c that showed the continuous pattern of up-regulation.

### Characterisation of two sporozoite surface proteins implicated in hepatocyte invasion

Antibodies were raised against a mixture of three polypeptides of 40 kDa that cover the entire PFD0425w protein (3 kb ORF with a predicted 113 kDa polypeptide with transmembrane domains), and against the PF08_0005 full recombinant protein (1.2 kb ORF encoding a predicted 45 kDa polypeptide with no transmembrane domains). Both proteins have a predicted signal peptide suggesting that they might be targeted to the secretory pathway and might be surface exposed. Western blotting was performed on pellets and supernatants from *P. falciparum* sporozoites incubated or not at 37°C.

**Figure 3:**
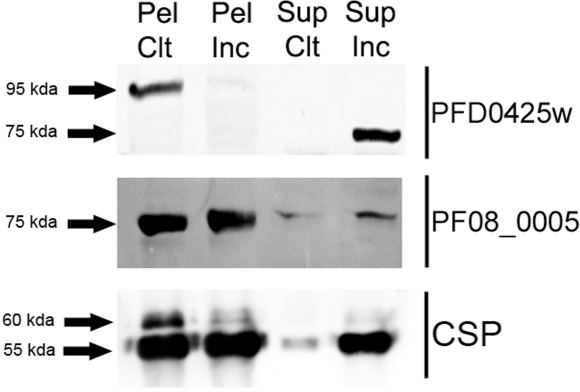
Detection of PFD0425w (SIAP-1) and PF08_0005 (SIAP-2) proteins in *P. falciparum* sporozoites. Western blots of pellets (Pel) or supernatants (Sup) from control salivary gland sporozoites (Ctl) or those incubated for 2 hours at 37°C (Inc), probed with both specific antisera or anti-PfCSP monoclonal antibody (lower panel). Arrows indicate the positions to which size markers had migrated.

Antibodies raised against the PFD0425w protein revealed a ≈95 kDa polypeptide in the pellet from control sporozoites (Figure 3) that was barely detectable in the pellet from 37°C-incubated sporozoites. By contrast, no reactivity was observed in the supernatant fraction from control sporozoites, whereas a ≈75 kDa band could be seen in that from the 37°C-incubated sporozoites. This pattern is reminiscent of AMA-1 and TRAP cleavage in sporozoites [25].

Antibodies raised against the PF08_0005 protein, revealed a ≈75 kDa protein in the pellets and supernatant fractions from both sporozoite preparations (Figure 3). The recombinant protein migrated at the 45 kDa level (data not shown), thus the higher size of the *in vivo* protein might be due to post-translational modifications. There was a clear though modest increase in the amount of shed protein in the supernatant of 37°C-incubated as compared to control sporozoites.

**Figure 4:**
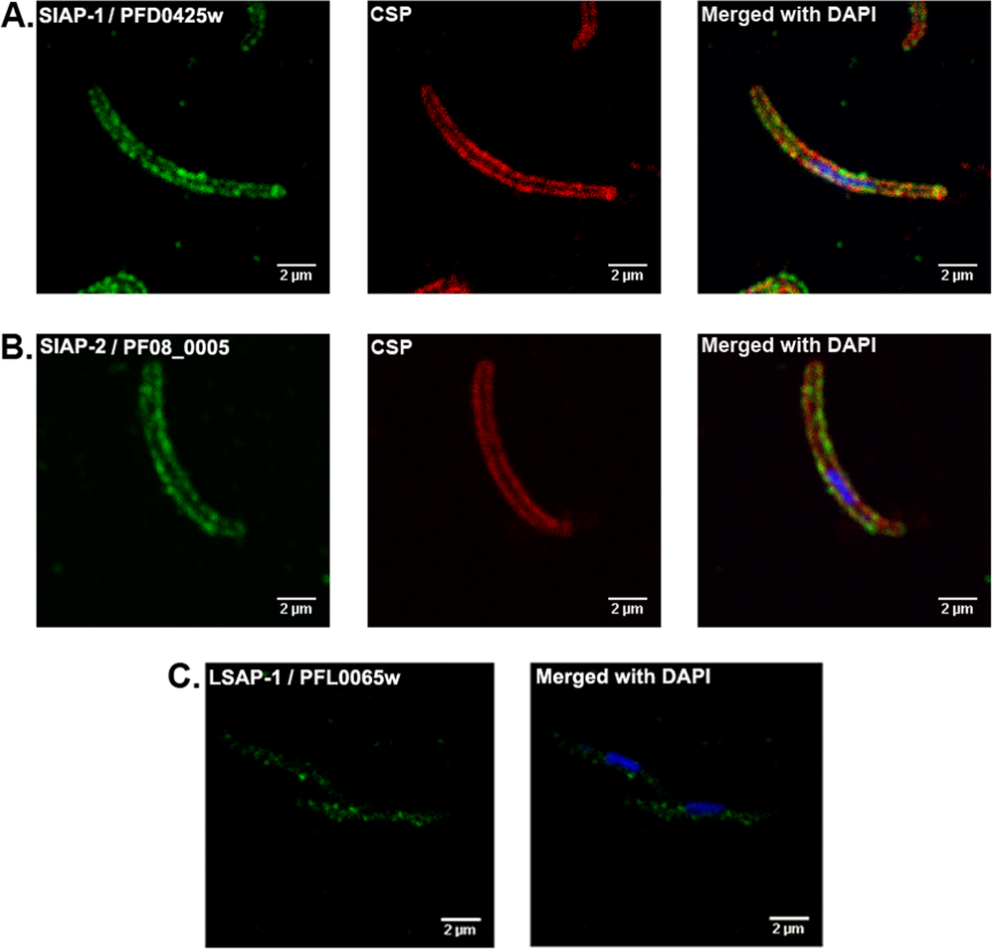
Localization of proteins encoded by genes up-regulated in *P. falciparum* sporozoites. Dual indirect IFA performed with a monoclonal antibody (red) targeting PfCSP, and anti-sera (green) specific to PFD0425w (SIAP-1) (Panel A), or PF08_0005 (SIAP-2) (Panel B). C, IFA on sporozoites with anti-PFL0065w (LSAP-1) serum (green). In all cases, sporozoite nuclei were visualized by DAPI staining (blue).

Localisation of the two proteins in the parasite was deduced from immunofluorescence assays (IFA) performed on salivary gland sporozoites. Specific staining for either protein revealed a strong peripheral and granular fluorescence similar in location and pattern to that obtained for CSP, suggesting localization on the sporozoite surface (Figure 4).

**Figure 5:**
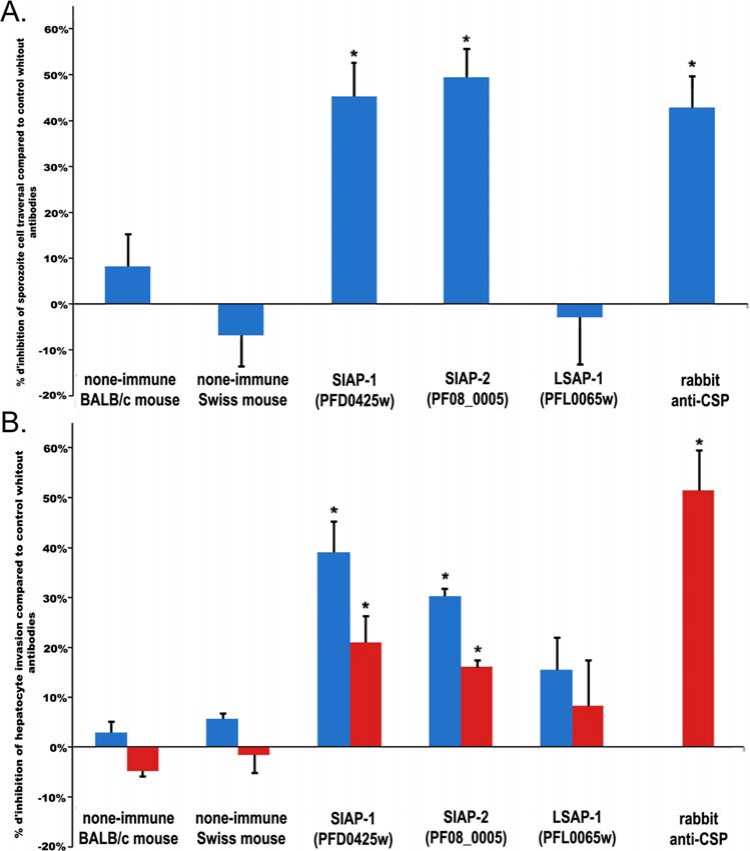
Inhibition assays of *P. falciparum* sporozoite cell traversal and human hepatocyte invasion. A. Inhibition of cell-traversal activity of *P. falciparum* sporozoites pre-incubated by different antisera (dilution 1/100). B. Inhibition of invasion of human hepatocytes by *P. falciparum* sporozoite *in vitro* by antisera to PFD0425w (SIAP-1), PF08_0005 (SIAP-2), or PFL0065w (LSAP-1) at two dilutions (1/20 blue or 1/100 red), or for rabbit anti-PfCSP serum (dilution 1/100 red) (2552±275.5 schizonts per control well for experiments using sera at 1/20 dilution, and 826±42 schizonts per well for those using sera at 1/100 dilution). For all experiments, inhibition was expressed as the means of the percentage of inhibition observed in two or more independent experiments, each performed in triplicate wells (+/−S.D.). * P<0.05 when compared to the control without antibodies.

The functional role of these proteins was explored by antibody inhibition assays. Pre-incubation of sporozoites with either of the two specific antibodies significantly decreased the percentage of cell traversal (Figure 5A). This inhibitory activity was comparable to that observed with anti-CSP serum at the 1∶100 dilution. The effect of the two antibodies on hepatocyte infection was then assessed using primary human hepatocytes to which *P. falciparum* sporozoites were added [34]. Again both antibodies led to a significant dose-dependent inhibition of invasion (Figure 5B), though inhibition was half that observed for anti-CSP serum at dilution of 1∶100.

In order to investigate the fate of the two proteins after hepatocyte invasion, IFA were performed on *P. falciparum*-infected primary human hepatocytes throughout the developmental stages (days 1 to 5 post-infection). Neither of the two proteins could be detected in liver stage parasites, including early forms (data not shown), strongly indicating that they were lost after hepatocyte invasion. Furthermore, when IFA and Western blots were performed on mixed blood stage parasites, no signal could be detected (data not shown), an observation consistent with the low levels of expression recorded in the blood stage parasite transcriptome [14],[35] and the absence from the blood stage proteome [18].

These observations are consistent with predominant expression in the sporozoite and a role in hepatocyte invasion. Thus, the proteins encoded by PFD0425w and PF08_0005 were named Sporozoite Invasion-Associated Protein-1 and -2 (SIAP-1 and SIAP-2), respectively. Orthologues of SIAP-1 were found in the genomes of *Plasmodium* species that infect rodents (*P. berghei*, *P. chabaudi* and *P. yoelii*) and primates (*P. reichenowi, P. vivax* and *P. knowlesi*), whereas SIAP-2 orthologues were only found in those that infect primates.

### Identification of two proteins predominantly expressed during the *Plasmodium* hepatic phase

Genes PFL0065w and PFB0105c encoded 2 proteins, with predicted transmembrane domains, of 12 kDa and 35 kDa, respectively. PFL0065w was characterized by the presence of a predicted signal peptide and PFB0105c by the presence of a PEXEL/VTS trafficking motif and a PHISTc domain [36]–[38]. Antibodies were raised against the full-length recombinant proteins and used as above in Western blots of sporozoites. No specific bands could be detected for the 2 proteins even when total extracts from 2×10^6^ sporozoites were probed (data not shown). When IFA were conducted on sporozoites, no reactivity was detected using anti-PFB0105c serum, but a weak internal punctuate staining was noted using anti-PFL0065w serum (Figure 4). Thus, it is likely that PFB0105c is not significantly expressed in sporozoites, whereas PFL0065w might. Nonetheless, anti-PFL0065w serum had no significant effect on sporozoite cell traversal, or on sporozoite invasion of primary human hepatocytes (Figure 5). These observations, suggested that the PFL0065w protein, if present in sporozoites, it would be so in small quantities and located internally.

**Figure 6:**
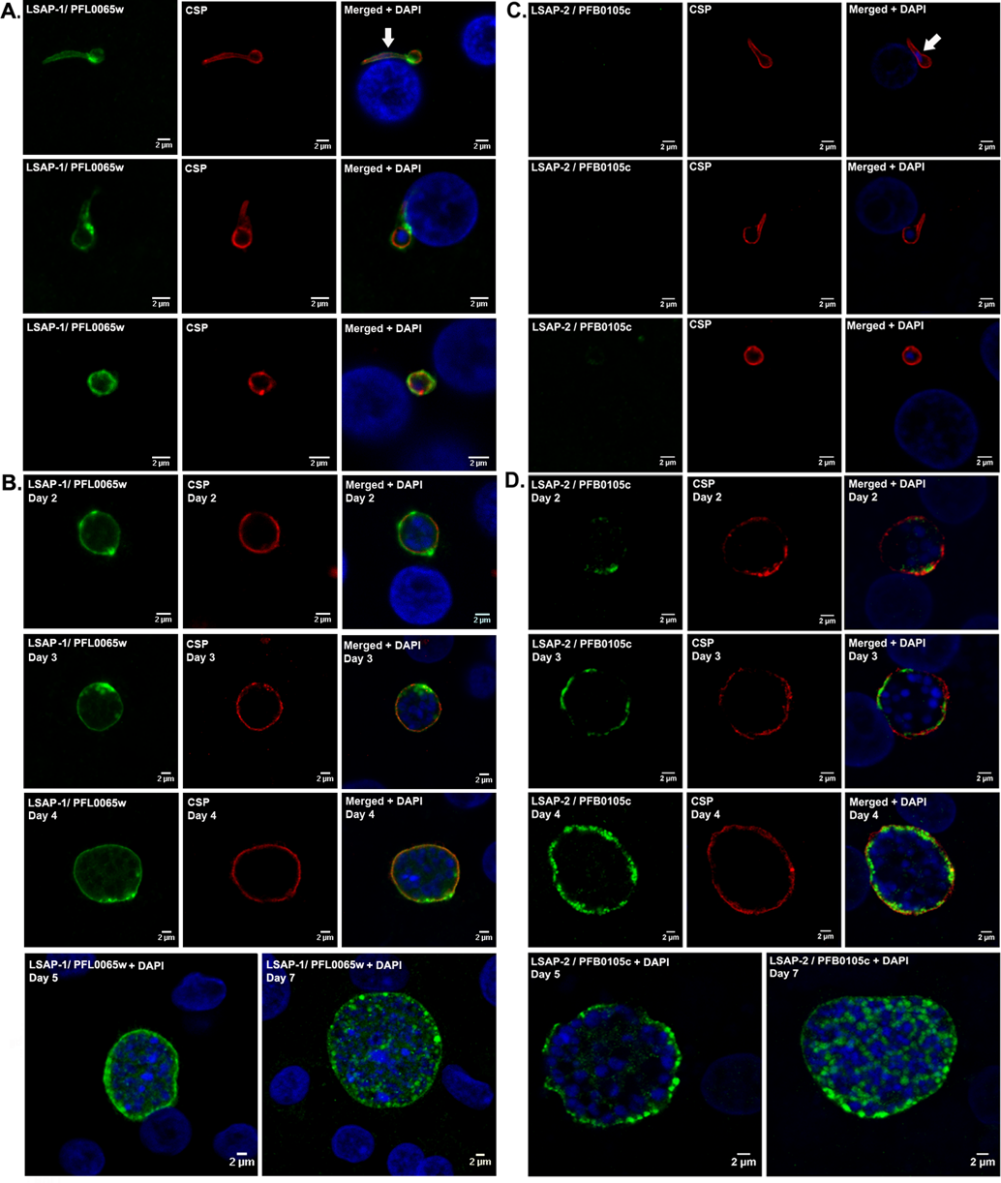
Temporal localization of PFL0065w (LSAP-1) and PFB0105c (LSAP-2) in liver stage parasites. Internal sections of cultured *P. falciparum* liver stage stained (green) with antisera specific to LSAP-1/PFL0065w (A and B) or to LSAP-2/PFB0105c (C and D) and (red) with antisera specific to PfCSP. Panels A and C correspond to *P. falciparum* sporozoites transforming into liver stage trophozoite during the first 24 hours after hepatocyte invasion, and panels B and D correspond to 2 up to 7 days old hepatic parasites Sporozoite nuclei stained with DAPI (blue) are indicated by a white arrow.

When IFA were performed on liver stage parasites, the PFL0065w protein was detected on transforming sporozoites (Figure 6A, day 1) within hepatocytes but not on those outside the hepatocyte (data not shown), and throughout liver stage development up to late schizont forms (Figure 6B day 7). By contrast, PFB0105c was detected only in older parasites, from trophozoite (2 days culture) up to the late schizont forms 7 days post-infection (Figure 6C and 5D). PFL0065w staining was strong, smooth and peripheral with one or several broad reinforcements observed within most exo-erythrocytic forms (EEF). The peripheral pattern overlapped extensively with the weak and discontinuous pattern observed for CSP, while in the regions of reinforcement PFL0065w staining appeared to lie outside the parasite surface with the fluorescence reaching into the hepatocyte cytoplasm (Figure 6B). PFB0105c staining displayed an uneven and peripheral pattern mostly co-localized with CSP with fluorescence reaching into the parasite cytoplasm and increasing in intensity with parasite age (Figure 6D). As the hepatic parasites matured, the PFL0065w staining pattern that was reminiscent of that previously observed for LSA-1 [39], suggested that the protein might be distributed around the developing schizonts (Figure 6B, day 5) before becoming confined around the cytomeres just before merozoite individualization (Figure 6B, day 7). It was interesting to observe that for PFB0105c, the staining in the maturing schizont was confined to granular areas with sharply defined edges between the nuclei of developing merozoites (Figure 6D, day 7).

No PFL0065w protein was detected by IFA or Western blots performed on mixed blood stage parasites (data not shown), whereas a moderate PFB0105c IFA signal was detected on mature erythrocytic parasites (Figure S1). These observations were consistent with data from the *P. falciparum* blood stages transcriptome [14],[35] and its blood and mosquito stages proteomes [18].

Taken together the above observations strongly suggest that PFL0065w and PFB0105c were mainly expressed in the liver stages of *P. falciparum*. These proteins were consequently named Liver Stage-Associated Protein -1 and -2 (LSAP-1 and LSAP-2, respectively). It was only possible to identify putative orthologue of LSAP-1 and LSAP-2 in the genomes of *Plasmodium* species that infect primates, *P. reichenowi*, *P. vivax* and *P. knowlesi* for LSAP-1, but only *P. vivax* for LSAP-2 [38].

## Discussion

*Plasmodium* sporozoites are abruptly subjected to the environment of the warm-blooded host often after extended periods of quiescence in the insect’s salivary gland lumen. In the mammalian host, the inoculated sporozoites are likely to remain extracellular for a few hours before infecting the hepatocytes, the only cell type where they can develop to maturity. We hypothesized that during this period, sporozoites are activated to a state of readiness for hepatocyte invasion. Parasite material corresponding to this transition period suitable for molecular investigations would be very difficult to obtain *in vivo*, especially if *P. falciparum* sporozoites are to be investigated.

We explored the influence of temperature and host cell contact, the two host environmental factors amenable to investigation *in vitro*, on the infectivity of *P. falciparum* sporozoite to hepatocytes [11]. We demonstrated that a temperature shift to 37°C is required to make salivary gland sporozoites infective to hepatocytes, though infectivity is lost within 30 minutes when only the sporozoites are incubated at this temperature. We showed that the loss in the *P. falciparum* sporozoites' infectivity can be prevented when incubation at 37°C is made in the presence of either of two types of human cells, skin keratinocytes or primary hepatocytes (Figure 1).

Recently, *in vivo* studies in rodents showed that the majority of sporozoites are deposited in the skin [4],[40] and migrate away from the site of inoculation over the next few hours [4]–[6]. One could speculate that residence in the mammalian host for a few hours is needed to bring the majority of the salivary sporozoites from a state of quiescence to that of hepatocyte-invasion readiness. Our observations performed *in vitro* with a human skin cell line or with human primary hepatocytes are consistent with this notion. The role of higher temperature (37°C) in optimal sporozoite infectivity might be simply explained in terms of the metabolic activation required to power motility. However, our results and previous studies indicate that this temperature shift has more wide-ranging consequences. First, sporozoites motility *per se* occurs without a temperature rise in the mosquito since the sporozoites released from the oocysts into the haemolymph migrate to and invade salivary gland sporozoites, albeit the mechanisms and speed of migration might differ from those in the mammalian host. Second, it was shown that a shift to 37°C activated exocytosis of *P. falciparum* sporozoite micronemes, a phenomenon associated with productive infection of hepatocytes, and this was enhanced when parasites were incubated with hepatocytes [25]. Third, a mere shift to 37°C, in the presence of serum, was sufficient to induce the transformation of *P. berghei* sporozoites into forms morphologically indistinguishable from early EEF [8]. Nonetheless, the importance of host cell contact was clearly demonstrated by the ability of hepatocytes to preserve sporozoite infectivity during lengthy incubation at 37°C (Figure 1).

We considered that the infectious sporozoites obtained after incubation in the co-cultures were physiologically similar to those found *in vivo* a few hours after the infectious mosquito bite. Thus, it was possible to obtain large numbers of viable and infectious sporozoites after incubation at 37°C in the presence of hepatocytes, so as to conduct whole transcriptome profiling analyses that would identify the modifications in steady-state levels of transcripts induced by the insect-to-mammalian host transition.

It was clear that exposure of salivary gland sporozoites to 37°C in the presence of hepatocytes for merely one hour, triggered complex and genome-wide changes in transcript levels. This substantial gene modulation probably participated to prepare, or possibly to activate, the sporozoite for successful infection. Indeed, the mRNA levels for 611 genes were altered in activated *P. falciparum* sporozoites, and for most genes (532/611) there was up-regulation. The genes identified included those that encode proteins involved in parasite transcription, translation, signalisation pathways, transportation, metabolism and also invasion. These observations are consistent with the concept that sporozoites that are ready to invade the hepatocyte productively are functionally different from the salivary gland sporozoites inoculated by the mosquito.

To date, little data is available on gene expression in *P. falciparum* pre-erythrocytic stages. Three genome wide expression data sets have become recently available for *P. yoelii* pre-erythrocytic stages: a cDNA library from sporozoites transformed axenically into early EEF forms, where 652 unique transcripts were identified [41]; a cDNA library of laser capture microdissected mature liver stages, where 623 unique transcripts were identified [42]; and most recently the transcriptome of liver stages at three points during their development, where 1985 actively transcribed genes were identified [43]. From a technical point of view, the data we present is most significantly comparable to that of Tarun *et al.* 2008 [43] since in both cases microarray analysis of the transcriptome was carried out, and changes in steady state levels were used as a criterion for gene identification. However, the data from Wang *et al.* 2004 [41], is biologically more relevant to our data, since it is derived from sporozoites that had been incubated at 37°C. Of the 611 *P. falciparum* genes identified in this study, 120 did not have orthologues in any of the three species that infect rodents according to orthology mapping data of Tarun *et al.* 2008 (see Figure S2 and Table S1). Of the remaining 491 *P. falciparum* genes with such orthologues, 321 were not represented in the sets identified in *P. yoelii* transformed sporozoites by Wang *et al.* 2004, or in the liver stage parasites used by Tarun *et al.* 2008. These relatively low levels of overlap are probably due to the fact that the data sets were derived from different stages: maturing hepatic stage parasites [43] or sporozoites incubated at 37°C for 24 hours in the absence of hepatocytes [41] as compared to the sporozoites incubated for 1 hour in the presence of hepatocytes from which our data was derived.

Confirmation that the microarray data presented reliably identified up-regulated genes was obtained through independent RT-qPCR analysis of a subset of 21 up-regulated genes. The RT-qPCR analysis further afforded the opportunity to investigate the time course of up-regulation over two hours of activation, and to explore the relative contribution of temperature or host cell contact on the alterations in gene expression. Two distinct patterns of up-regulation were observed (Figure 2). It was hypothesised that these patterns can provide an indication as to the likely role of the corresponding proteins. The modest transient up-regulation observed following incubation at 37°C in the presence of hepatocytes was characteristic for the selected genes known to be associated with invasive processes. In this case, it would appear that contact with hepatocytes alone accounted for the observed up-regulation (Figure 2), since no up-regulation was observed when the sporozoites were incubated at 37°C without hepatocytes. For the group of genes that included those encoding proteins known or likely to be expressed during hepatic stage development, up-regulation increased continuously with the duration of incubation at 37°C in the presence of hepatocytes, and the levels reached tended to be high. In this case, the shift in temperature seemed to be the effective signal (Figure 2), since similar changes in expression were observed irrespective of the contact with hepatocytes. At this stage, it is not possible to conclude that these increases translate into higher protein levels. Although global analysis of protein and RNA levels showed a positive correlation between mRNA and protein abundance, delays between mRNA and protein accumulation were noted for many genes [44]. This issue can only be addressed by analysis of individual genes and their products.

Functional studies of two hypothetical proteins encoded by genes with the transient pattern of up-regulation, confirmed that they were likely to be implicated in host cell invasion. IFA staining patterns suggested that SIAP-1 and SIAP-2 were located at the surface of the sporozoite. Western blotting showed that they were released in the supernatant upon incubation at 37°C, and that SIAP-1 was cleaved after release. Further studies are required to determine whether cleavage is achieved using the same proteolytic machinery implicated in the shedding of TRAP and AMA-1 [25]. Cell invasion inhibition assays showed that both anti-SIAP-1 and anti-SIAP-2 blocked sporozoite migration and productive invasion of hepatocytes, further supporting surface localization. The fact that inhibition levels for cell traversal were similar to those afforded by anti-PfCSP antibodies, while those for hepatocyte invasion were lower (in particular for SIAP-2) suggest that SIAP-1 and SIAP-2 might have a more prominent role in sporozoite gliding than in hepatocyte invasion. Future studies would require inhibitory activities to be measured in terms of the quantity of specific antibodies rather than in terms of dilution to evaluate the potential of these 2 proteins as vaccine candidates. It should be noted that the *P. yoelii* orthologue of SIAP-1 was detected in the proteome of the mature liver stages of this parasite [43], but not in that of *P. berghei* parasites. It is not clear at present whether the IFA negative results obtained for *P. falciparum* liver stage reflect the absence of SIAP-1, post-translational modifications that adversely affect antibody recognition, or species differences. The modest increases noted for transiently up-regulated genes is consistent with proteins associated with motility and/or invasion, because these proteins are likely to be already present in salivary gland sporozoites, as is the case for TRAP and aldolase that participate in the motor machinery powering gliding motility [24], [30], [45]–[47]. At best, the transient up-regulation of invasion-related genes might serve to compensate for the loss of invasive proteins shed by the parasite during migration to its final target cell.

Characterization of the two hypothetical proteins encoded by genes with the continuous pattern of up-regulation showed that they were expressed throughout hepatic parasite development. The two corresponding genes had reached high expression levels as a result of activation. Detectable increases in LSAP-1 protein levels were noted as early as day 1 post-inoculation in transforming sporozoites, whereas LSAP-2 protein could only be detected from day 2 post inoculation. LSAP-1 was mainly found at the periphery of the intracellular hepatic parasite throughout its development, but not in blood stage parasites and possibly in minor quantities in salivary gland sporozoites. Thus, LSAP-1 might represent the second liver stage-specific protein to be identified in *P. falciparum*, the other one being LSA-1 [48]. LSAP-2 was also mainly expressed at the periphery of the intracellular hepatic parasite, but it was additionally detectable in moderate quantities in blood stage parasite though not in salivary gland sporozoites. The presences of a PEXEL/VTS domain in LSAP-2 and of a signal peptide in LSAP-1 are consistent with location at the PVM and eventual export to the host cell cytoplasm, though the proteins were not detected in the cytoplasm of infected hepatocytes. Knock-out studies, such as those used for UIS3 [49], UIS4 [20] and P36p [50] will be required to ascribe a function for these protein or to establish whether they are essential for liver stage development. It is interesting to note that there were no identifiable orthologues for either gene in the rodent model species, as was the case for SIAP-2 and LSA-1, which suggests that they might interact with components specific to the primate hepatocyte.

In conclusion, whole genome transcriptome analysis of *P. falciparum* sporozoite activated by relatively brief exposure to mammalian host-like conditions, revealed major changes in gene expression. A fifth of genes identified as being up-regulated in activated *P. falciparum* sporozoites had no orthologues in the genomes of the *Plasmodium* species used as experimental models in rodents. Some of the proteins encoded by these genes might have specifically evolved to optimise the interactions between the parasite and the human liver. The data presented provides a gateway for the identification of new *P. falciparum* genes implicated in the processes of hepatocyte invasion and liver stage development, and as such some of these might prove valuable as potential vaccine targets. In a previous study, 16 *P. falciparum* sporozoite proteins were identified as being highly antigenic based on stimulation of immune cells obtained from volunteers immunized with radiation-attenuated sporozoites [31]. Five of these proteins including SIAP-1 were encoded by genes up-regulated in the activated sporozoite transcriptome (Table S1). It is hoped that future studies might provide the basis for unravelling the biology of, and eventually elaborate therapies against, the few short-lived stages that occur between the bite of an infected mosquito and the release of merozoites, which constitute the obligatory steps without which the establishment of a *Plasmodium* infection cannot occur.

## Materials and Methods

### Isolation of *P. falciparum* sporozoites

Adult *Anopheles stephensi* females were infected with the NF54 strain of *P. falciparum* [51]. After 14–21 days, the salivary glands were aseptically dissected and sporozoites were purified over a 27% then a 75% Percoll gradient and kept at 4°C.

### Isolation and culture of human hepatocytes

Primary human hepatocytes were isolated from human liver fragments, collected during unrelated surgery after informed consent and in agreement with French national ethical regulations, and were cultivated as described elsewhere [25]. Hepatocytes were seeded in 24-well culture plates (5×10^5^ cells/well) for incubation with sporozoites, in eight-chamber plastic Lab-Tek slides (2.1×10^5^ cells/well) for *in vitro* culture of liver stage parasite, or in 6-well culture plates (2.5×10^6^ cells/well) for *P. falciparum* sporozoite transcriptome analyses.

### Standard sporozoites infectivity assay

Human hepatocytes were cultured for at least 24 hour before inoculation with *P. falciparum* sporozoites. After the removal of medium from the culture chambers, sporozoites in culture medium were added to the Lab-Tek wells (1×10^5^ sporozoites/well). After 3 hours at room temperature (for sporozoite sedimentation) and 3 hours at 37°C (for sporozoite invasion), the cultures were washed to remove sporozoites that have not penetrated hepatocytes, and then incubated at 37°C in fresh medium for 3 days to obtain liver schizonts. Hepatic cultures were then fixed in methanol and stained using an anti-HSP-70 mouse serum followed by goat anti-mouse Alexa 494 conjugate (Invitrogen, Paisley, United Kingdom) and counted under a fluorescence microscope. Typically, 300 to 1000 hepatic parasites are obtained per well. Infectivity was expressed as the percentage of the number of schizonts observed as compared to that obtained in controls where salivary gland sporozoites were used.

The importance of the temperature shift on sporozoite infectivity was determined by incubating the sporozoites with hepatocytes for the whole 6 hours at room temperature prior to washing and subsequent incubation for 3 days at 37°C.

### Influence of temperature and host-cell contact on the infectivity and the cell-traversal activity of *P. falciparum* sporozoites

The infectivity of sporozoites subjected to different conditions (data presented in Figure 1) was assessed using the standard sporozoite infectivity assay. Salivary gland sporozoites were added to 24-well culture plates that were seeded or not with human primary hepatocytes or human skin keratinocytes (HaCaT cell line, a gift from Dr Alain Simon, Faculty of Pharmacy, Limoges, France). The plates were then centrifuges (1800×g for 5 min) at room temperature to ensure optimal contact with the host cells, and then incubated for 30 minutes, 1 hour, or 2 hours at 37°C. At the end of the incubation period, the sporozoites that had not invaded or irreversibly attached were recovered by washing and counted (80% recovery was generally obtained). The infectivity of these sporozoites was then tested by the standard sporozoites infectivity assay (see above).

Cell-traversal activity of sporozoites was assessed as described previously with slight modifications [2]. Sporozoites (3.0×10^5^) were added to HeLa cells seeded on a 96-well plate. The plate was centrifuged to spin down the sporozoites and then incubated for 3 hours in the presence of 0.5 mg/ml FITC-dextran 10000 MW (Invitrogen, Paisley, United Kingdom) at 37°C. The cells were then washed, trypsinized, and resuspended with 1% formaldehyde in PBS. The percentage of wounded cells (FITC-positive) was determined by FACS.

### Microarray protocol

Activated sporozoites were obtained as follows: 40 millions *P. falciparum* salivary gland sporozoites were added to human primary hepatocytes cultures, and the plates centrifuged (1800×g for 5 min) at room temperature to ensure optimal contact with the host cells. The plates were then incubated at 37°C for 1 hour, at the end of which unattached sporozoites were recovered by washing (>80% recovery) and enumerated. An equivalent number of salivary gland sporozoites (to the ones recovered above) were used as control sporozoites. RNA from both sporozoite populations was purified using an RNeasy micro kit (Qiagen, Hilden, Germany). [^3^H]-radiolabelled cDNA, synthesized from 0.25 µg total RNA [17], was hybridized to a pan-genomic DNA microarray containing 12037 unique 70-mer oligonucleotides [13], in the presence of 5 µg of total RNA purified from uninfected mosquitoes. Acquisition of the arrays was carried out as described previously [52]. Three biological replicates for each condition were performed and data were normalized according to the median value of the total intensities of all spots. Background was defined as the average value of the control spots without oligonucleotides. Genes with an intensity >3 fold the background (the average of three independent experiments) either in the sporozoites incubated with hepatocytes or in the salivary gland sporozoites conditions, and with a two-fold or more change in expression between the two types were considered to be up- or down-regulated.

### Multiplex quantitative PCR

Forty millions control sporozoites and the same number of sporozoites added to culture plates and then centrifuged before incubation at 37°C in the presence or in the absence of human hepatocytes were processed for RNA extraction. Total RNA was treated with DNase and then reverse transcribed. TaqMan PCR data was derived from two independent experiments each performed in triplicate using the cDNA produced from 10 ng of total RNA [17]. None of the primers and probes (Table S2) cross-reacted with mosquito or human DNA (data not shown). Control reactions were performed without reverse transcriptase to verify the absence of genomic DNA contamination (data not shown). Quantitative gene expression data were normalized with respect to the parasite's 18S RNA cDNA levels and relative gene expression was expressed via the log_2_ of the ratio expression using the 2_T_^−ΔΔC^ method [53].

Since several hours were needed for the dissection of the 1200 mosquitoes required to gather the 80 million sporozoites used for transcriptome profiling, it was necessary to store them at 4°C until incubation at 37°C, because storage at room temperature for extended periods would have led to a loss in motility and infectivity [7]. In order to confirm that storage of sporozoites on ice for many hours did not influence the pattern of up-regulation observed, RT-qPCR was conducted for a subset of 13 out of the 21 genes included in the RT-qPCR analysis. Sporozoite samples were kept at 4°C for 2 hours before incubation at 37°C with hepatocytes, or were directly incubated after dissection at room temperature. No differences in the levels of up-regulation were noted between the two groups of sporozoites (Table S3).

### Recombinant protein expression and antisera production

DNA fragments corresponding to coding sequences of SIAP-1 (PFD0425w), SIAP-2 (PF08_0005), LSAP-1 (PFL0065w) and LSAP-2 (PFB0105c) were PCR amplified from 3D7 *P. falciparum* genomic DNA and inserted into a pEXP5-Nt/TOPO or pEXP5-Ct/TOPO plasmid (Invitrogen, Paisley, United Kingdom), both His-Tag expression vectors. Recombinant proteins were expressed using an Active-pro *in vitro* translation kit (Ambion) and purified with Ni-Sepharose-6-Fast-Flow beads (GE Healthcare, Buckinghamshire, United Kingdom) following the manufacturer's batch protocol. His-Tagged proteins linked to Ni-Sepharose-6-Fast-Flow beads were washed with Phosphate-buffered saline (PBS) and injected intra-peritoneally into female BALB/C or Swiss mice at 2 week interval. One week after the third immunization, blood was collected to prepare serum. Specificity of the anti-sera, from BALB/C (SIAP-1, SIAP-2, LSAP-2 sera) or Swiss (LSAP-1 serum) mice, was confirmed by probing Western blots of the recombinant proteins (data not shown).

### Western blotting and secretion assay

Purified sporozoites were equally divided and suspended in medium without serum and incubated for 2 hours either at 4°C or 37°C. Supernatants and sporozoite pellets, obtained after centrifugation at 16,000×*g* for 3 minutes, were dissolved in Laemmli buffer and incubated at 90°C for 5 minutes, before being subjected to 10% -15% SDS-PAGE (1×10^6^ parasites/lane for SIAP detection, and 3×10^4^ parasites/lane for CSP detection) and transferred onto nitrocellulose membranes. These were incubated for 1 hour with Odyssey blocking solution (Li-cor, Lincoln, USA) and probed overnight at 4°C with primary antibody [dilution 1/500 for anti-SIAP sera and 1/15000 for monoclonal E9 anti-CSP antibodies [54]]. Membranes were then incubated for 1 hour at room temperature with an IRDye 800CW Goat Anti-Mouse IgG secondary antibody (Li-cor, Lincoln, USA) and immuno-stained proteins were visualized using the Odyssey infrared imaging system (Li-cor, Lincoln, USA). Non-infected salivary glands and *E. coli* protein extracts were used as negative controls (data not shown). All Western blots were performed at least twice using extracts from two independent experiments.

### Immunofluorescence assays

Air-dried *P. falciparum* sporozoites, permeabilized with 0.1% triton X100 and blocked with 3% BSA in PBS, were incubated with mouse anti-SIAP and anti LSAP sera (dilution 1/200), followed by incubation with anti-mouse Alexa Fluor 494 (Invitrogen, Paisley, United Kingdom), and 1 µg/ml DNA stain diamidino-phenylindole (DAPI). Non-immune mouse serum was used as control. Dual staining with anti-CSP was performed using rabbit anti-CSP serum (1/7000) (a gift from Dr Laurent Rénia, Cochin Institute, Paris, France) using anti-rabbit IgG Alexa Fluor 594 (Invitrogen, Paisley, United Kingdom) as a secondary antibody. Liver stage staining was performed on hepatocyte cultures fixed with 4% paraformaldehyde and then permeabilized with methanol. Slides were then examined by confocal fluorescence microscopy.

### In vitro inhibition of sporozoite cell-traversal and invasion

In order to quantify the inhibitory potential of antisera on sporozoite cell-traversal activity, sporozoites (3.0×10^5^) incubated in the presence or the absence of antisera (dilution 1/100) were added to HeLa cells seeded on a 96-well plate, and the cell-traversal assay carried out as described above. The extent of inhibition was expressed as the percent ratio of the percentage of wounded cells observed in the presence of antibody over that observed in the absence of antibody. To determine the effects of antisera on sporozoite invasion ability, triplicate hepatocyte cultures were inoculated with *P. falciparum* sporozoites (1.0×10^5^/Lab-Tek well) incubated with anti-SIAP or anti-LASP (dilution 1/20 or 1/100), anti-CSP (dilution 1/100), or non-immune mice sera. Liver stage parasite were cultivated and stained as described above. The extent of inhibition was expressed as a percentage calculated as the number of schizonts in wells exposed to antibody over that in control wells.

### Gene orthology mapping

Orthologues of *P. falciparum* SIAP and LSAP genes were sought in the genomes of *Plasmodium* species that infect rodents and primates: *P. yoelii, P. berghei, and P. chabaudi, P vivax, and P knowlesi* (BLAST program available at www.PlasmoDB.org), and *P. reichenowi* (TBLASTN program available at http://www.sanger.ac.uk/cgi-bin/blast/submitblast/p_reichenowi). Orthologous sequences from two genomes were defined as reciprocal best hits with BLAST E-values less than 1e-15. Failure to detect orthologues for SIAP and LSAP proteins with program based on protein sequence homology, was confirmed by additional analyses based on gene synteny maps [55].

### Statistical analysis

Data obtained after functional assays were analyzed for statistical significance using the one-way analysis of variance followed by the Tukey multiple comparison test.

### *P. falciparum* genes ID (PlasmoDB 5.4) mentioned in the manuscript and potential annotation of the corresponding proteins in *Plasmodium* species infecting human and rodents

PFD0425w : SIAP-1

PF08_0005 : SIAP-2

PFL0065w : LSAP-1

PFB0105c : LSAP-2

PF13_0201 : TRAP

PF14_0425 : fructose-bisphosphate aldolase

PF11_0344 : AMA-1

PFB0570w : SPATR

MAL13P1.212 : SPECT-1

PFD0430c : SPECT-2

PF08_0054 : heat shock 70 kDa protein

PFC0210c : Circumsporozoite (CS) protein

PFL0800c : CelTOS

PF10_0164 : UIS-4

PF13_0012 : UIS-3

PF11_0224 : EXP-1

PF14_0678 : EXP-2

MAL8P1.6 : Etramp 8

MAL6P1.135 : Phospholipase

PF08_0099 :P36p

## Supporting Information

Figure S1 Localization of PFB0105c (LSAP-2) in mature blood stage culture. Internal section of *P. falciparum* schizont and merozoites (white arrows) stained with anti-PFB0105c sera (green) and DAPI (Blue).
(0.08 MB PDF)

Figure S2 Venn diagram of the genes identified by selected transcriptome analysis of pre-erythrocytic parasites. The data derived from *P. falciparum* (this work) was compared with that obtained from *P. yoelii* using axenically transformed sporozoites (Wang et al. 2004) and using different developmental stages of hepatic parasites (Tarun et al. 2008). Only *P. yoelii* genes with orthologues in P. falciparum were considered for this analysis. It should be noted that the low proportion of genes with orthologues in *P. falciparum* (22% i.e. 146 genes) in transforming sporozoites was probably an underestimate due to the nature of the genomic datasets available at that time. Of the 1985 genes identified by Tarun et al. 2008, 66% (1305 genes) had orthologues in *P. falciparum*. This value closely reflects that obtained when the genome of *P. falciparum* is compared to that of the three *Plasmodium* species that infect rodents, *P. chabaudi, P. berghei* and *P. yoelii* (Hall et al. 2005; Tarun et al. 2008).
(0.09 MB DOC)

Table S1 Microarrays Results.
(0.05 MB PDF)

Table S2 Primers used for Taqman RT-qPCR experiments.
(0.01 MB PDF)

Table S3 Influence of temperature storage of sporozoite on the pattern of a number of up-regulated genes.
(0.08 MB PDF)
