# Supplementary material for: Temperature Shift and Host Cell Contact Up-Regulate Sporozoite Expression of Plasmodium falciparum Genes Involved in Hepatocyte Infection
Source: PLoS Pathog. 2008 Aug 8;4(8):e1000121. doi: 10.1371/journal.ppat.1000121 (PMC2488394; doi:10.1371/journal.ppat.1000121)
Supplement: Figure S1 — Localization of PFB0105c (LSAP-2) in mature blood stage culture. Internal section of P. falciparum schizont and merozoites (white arrows) stained with anti-PFB0105c sera (green) and DAPI (Blue). (0.08 MB PDF) [file ppat.1000121.s001.pdf]

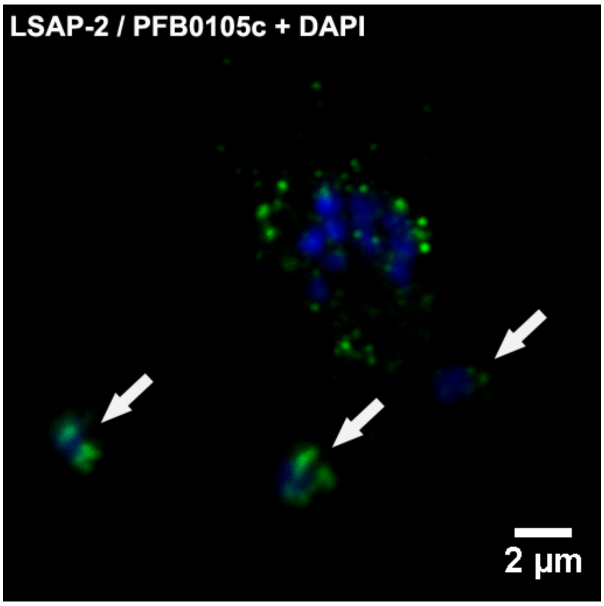

Figure S1. Localization of PFB0105c (LSAP-2) in mature blood stage culture. Internal section of *P. falciparum* schizont and merozoites (white arrows) stained with anti-PFB0105c sera (green) and DAPI (Blue).
