# Supplementary material for: Temperature Shift and Host Cell Contact Up-Regulate Sporozoite Expression of Plasmodium falciparum Genes Involved in Hepatocyte Infection
Source: PLoS Pathog. 2008 Aug 8;4(8):e1000121. doi: 10.1371/journal.ppat.1000121 (PMC2488394; doi:10.1371/journal.ppat.1000121)
Supplement: Figure S2 — Venn diagram of the genes identified by selected transcriptome analysis of pre-erythrocytic parasites. The data derived from P. falciparum (this work) was compared with that obtained from P. yoelii using axenically transformed sporozoites (Wang et al. 2004) and using different developmental stages of hepatic parasites (Tarun et al. 2008). Only P. yoelii genes with orthologues in P. falciparum were considered for this analysis. It should be noted that the low proportion of genes with orthologues in P. falciparum (22% i.e. 146 genes) in transforming sporozoites was probably an underestimate due to the nature of the genomic datasets available at that time. Of the 1985 genes identified by Tarun et al. 2008, 66% (1305 genes) had orthologues in P. falciparum. This value closely reflects that obtained when the genome of P. falciparum is compared to that of the three Plasmodium species that infect rodents, P. chabaudi, P. berghei and P. yoelii (Hall et al. 2005; Tarun et al. 2008). (0.09 MB DOC) [file ppat.1000121.s002.doc]

**
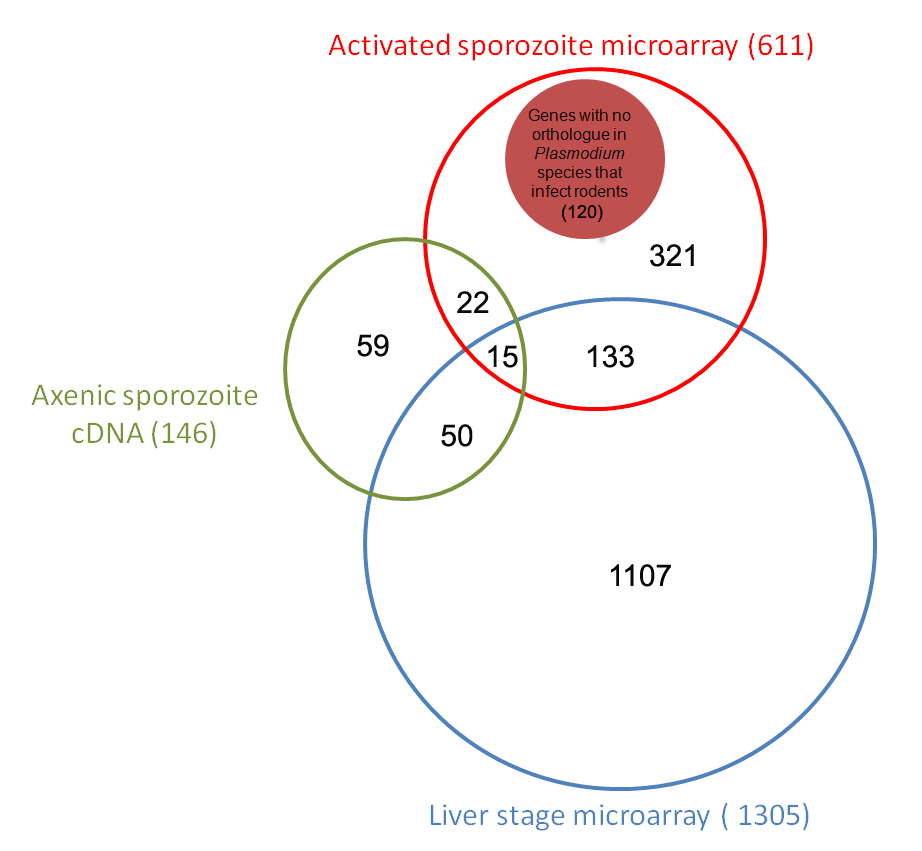
**

**Figure S2. Venn diagram of the genes identified by selected transcriptome analysis of pre-erythrocytic parasites.** The data derived from *P. falciparum* (this work) was compared with that obtained from *P. yoelii* using axenically transformed sporozoites [41] and using different developmental stages of hepatic parasites [43]. Only *P. yoelii* genes with orthologues in P. falciparum were considered for this analysis. It should be noted that the low proportion of genes with orthologues in *P. falciparum* (22% i.e. 146 genes) in transforming sporozoites was probably an underestimate due to the nature of the genomic datasets available at that time. Of the 1985 genes identified by Tarun et al. 2008, 66% (1305 genes) had orthologues in *P. falciparum*. This value closely reflects that obtained when the genome of *P. falciparum* is compared to that of the three *Plasmodium* species that infect rodents, *P. chabaudi*, *P. berghei* and *P. yoelii*, [10,43].
