# Supplementary material for: Temperature Shift and Host Cell Contact Up-Regulate Sporozoite Expression of Plasmodium falciparum Genes Involved in Hepatocyte Infection
Source: PLoS Pathog. 2008 Aug 8;4(8):e1000121. doi: 10.1371/journal.ppat.1000121 (PMC2488394; doi:10.1371/journal.ppat.1000121)
Supplement: Table S1 — Microarrays Results. (0.05 MB PDF) [file ppat.1000121.s003.pdf]

Table S1. Microarrays Results

GENES UP-REGULATED IN SPOROZOITES INCUBATED AT 37°C WITH HEPATOCYTES

gene.ID	salivary Gland sporozoite mean intensity	sporozoite incubated 1 hr at 37C° with hepatocytes mean intensity	expression ratio (sporozoite incubated 1 hr at 37C° with hepatocytes / salivary gland sporozoite)	Found in Axenic liver stage (Wang et al 2004)	contains a predicted signal peptide/anchor (SignalP)	Contains at least 1 predicted transmembrane domains (TMAP, TMPRED, TOPPRED2 and TMHMM2)	detected in sporozoite using mass spectrometry (Florens et al 2002)	Presence of orthologue in *P. berghei* (tarun et al 2008)	Presence of orthologue in *P. chabaudi* (tarun et al 2008)	Presence of orthologue in *P. yoelii* (tarun et al 2008)	description
**host cell invasion /cell passage**											
PF08_0005	250	2544	10,2		yes		yes	NA	NA	NA	SIAP-2
PF13_0201	1355	9509	7,0	yes	yes	yes	yes	PB000374.03.0	PC103922.00.0	PY03052	TRAP/ sporozoite surface protein 2
PFD0425w	481	2111	4,4		yes	yes	yes	PB000251.01.0	PC000627.01.0	PY00455	SIAP-1 /S5 (Kaiser et al 2004)/ protein recongnized by volunteers immunized by irradied*P.falciparum* sporozoite (Doolan et al 2003)
PF14_0425	241	978	4,1			yes	yes	PB000757.02.0	PC001392.02.0	PY03709	fructose-bisphosphate aldolase (Buscaglia et al 2003)
PFF1420w	387	1379	3,6		yes	yes		PB000484.00.0	PC000649.03.0	PY04170	phosphatidylcholine-sterol acyltransferase precursor putative (Bhanot et al 2005) / UIS10 (Matuschewski et al 2002)
PF11_0344	406	1354	3,3		yes	yes	yes	PB000821.01.0	PC301665.00.0	PY01581	apical membrane antigen 1 precursor (Silvie et al 2004)
PFB0570w	286	916	3,2		yes			PB001140.00.0	PC000225.03.0	PY02991	pfSPATR (Chattopadhyay et al 2003)
PF10_0281	225	727	3,2		yes	yes		PB000355.03.0	PC000113.04.0	NA	merozoite TRAP-like protein, MTRAP (Baum J et al 2005)
PFF0800w	341	1053	3,1		yes	yes		PB001517.02.0	PC000366.04.0	PY01499	TRAP-like protein, TRAP2 (Baum J et al 2005)
PFD0430c	728	2138	2,9	yes	yes	yes		PB000252.01.0	PC000807.02.0	PY00454	SPECT-2 (Ishino et al 2005)
MAL13P1.212	1027	2960	2,9		yes			NA	NA	NA	SPECT-1 (Ishino et al 2004)
PF14_0102	421	958	2,3		yes		yes	PB000779.00.0	PC000272.01.0	PY00622	rhoptry-associated protein 1, RAP1 (Harnyuttanakorn et al 1992)
PF14_0495	424	862	2,0		yes	yes	yes	PB000379.02.0	PC000433.04.0	PY06813	rhoptry neck protein 2, putative
**metal-ion homeostasis**											
PFF1325c	350	1594	4,6			yes		PB000069.01.0	PC000488.03.0	PY04641	c3h4-type ring finger protein putative
PF13_0019	214	806	3,8			yes		PB000105.00.0	PC000865.01.0	PY02931	sodiumhydrogen exchanger, Na+, H+ antiporter
PF14_0518	431	1414	3,3			yes		PB000632.00.0	PC000972.01.0	PY00856	nifU protein putative
**metabolic processes**											
PFL1260w	261	2214	8,5			yes		PB001058.01.0	PC000009.01.0	PY03530	hydrolase phosphatase, putative
PFI1090w	379	2690	7,1			yes		PB000151.03.0	PC000567.01.0	PY06246	s-adenosylmethionine synthetase, putative
PF10_0322	485	3139	6,5			yes	yes	PB000516.03.0	PC000866.02.0	PY04754	S-adenosylmethionine decarboxylase-ornithine decarboxylase
PF13_0286	436	2821	6,5	yes		yes	yes	PB000001.01.0	PC000023.01.0	PY02347	methyltransferase, putative
PF14_0447	1936	11823	6,1	yes		yes		PB000083.02.0	PC000109.02.0	PY06896	glutaminyl-peptide cyclotransferase
PFC0050c	1162	4825	4,2		yes	yes		NA	NA	NA	long chain acyl-CoA synthase, ATP dependent, family 1, isoform 6
MAL7P1.150	296	1223	4,1					PB000702.00.0	PC000890.01.0	PY02096	cysteine desulfurase, putative
PF10_0218	317	1216	3,8			yes	yes	PB001560.02.0	PC302082.00.0	PY01660	citrate synthase, mitochondrial precursor, putative
MAL8P1.156	1166	3998	3,4			yes	yes	PB000906.01.0	PC000941.01.0	PY03463	mannose-6-phosphate isomerase, putative
PFD0285c	271	921	3,4			yes		PB300815.00.0	PC000176.03.0	PY00349	lysine decarboxylase, putative
MAL8P1.17	237	797	3,4		yes		yes	PB001298.00.0	PC000663.04.0	PY00638	disulfide isomerase precursor, putative
MAL13P1.121	1108	3738	3,4		yes	yes		PB000368.00.0	PC000035.00.0	PY06466	adenosine-diphosphatase
PF14_0615	289	924	3,2				yes	PB300802.00.0	PC000362.04.0	PY04403	ATP synthase (CAC39) subunit, putative
PFI1370c	356	1079	3,0			yes		PB001578.02.0	PC000153.02.0	PY05588	phosphatidylserine decarboxylase, PfPSD
PFI0945w	166	489	2,9	yes			yes	PB000418.03.0	PC000932.01.0	PY05336	Thioredoxin, putative
PFL1270w	175	510	2,9				yes	PB001056.01.0	PC000833.02.0	PY03531	cof-like hydrolase, had-superfamily, subfamily iib
PF14_0164	241	663	2,8			yes		PB300230.00.0	PC000975.01.0	PY04261	NADP-specific glutamate dehydrogenase
PFF0625w	244	647	2,7			yes		PB000526.00.0	PC000909.01.0	PY03098	nucleolar GTP-binding protein 1, putative
PFE0700c	904	2376	2,6			yes		PB000310.03.0	PC000726.01.0	PY02818	tubulin-tyrosine ligase, putative
PFF0530w	365	962	2,6			yes	yes	PB001252.02.0	PC000215.04.0	PY03111	transketolase putative
PFI1670c	171	440	2,6					PB001133.02.0	PC000234.01.0	PY06101	vacuolar ATP synthase subunit E, putative
MAL13P1.319	235	609	2,6		yes	yes	yes	PB000510.01.0	PC000325.03.0	PY00839	indole-3-glycerol-phosphate synthase, putative
PF14_0598	179	443	2,5			yes	yes	PB000084.03.0	PC000143.05.0	PY03280	glyceraldehyde-3-phosphate dehydrogenase
PFE0555w	505	1245	2,5			yes		PB000912.01.0	PC000036.02.0	PY04895	stearoyl-CoA Delta 9 desaturase, putative
PF11_0405	298	705	2,4				yes	PB000439.01.0	PC000026.04.0	PY02067	AAA family ATPase, putative
PFI1110w	178	430	2,4			yes		PB000319.00.0	PC000947.01.0	PY04688	glutamate--ammonia ligase (glutamine synthetase), putative
PF14_0038	275	657	2,4					PB001331.02.0	PC000171.05.0	PY05430	cytochrome c, putative
PFL0630w	235	558	2,4			yes	yes	PB000191.02.0	PC000220.05.0	PY04921	iron-sulfur subunit of succinate dehydrogenase
MAL13P1.225	304	699	2,3		yes	yes		PB105583.00.0	PC103721.00.0	PY00586	thioredoxin, putative
PF08_0113	460	1054	2,3			yes	yes	PB000782.00.0	PC300005.00.0	PY05420	vacuolar proton-translocating ATPase subunit A, putative
PF11_0412	202	456	2,3			yes		PB000656.03.0	PC301156.00.0	PY00743	Vacuolar ATP synthase subunit F, putative
PF14_0660	188	406	2,2		yes	yes		PB000392.02.0	PC302368.00.0	PY03645	phosphoesterase, putative
PF14_0314	181	396	2,2			yes		PB000212.01.0	PC000775.03.0	PY05679	chromatin assembly factor 1 p55 subunit, putative
PF10_0274	252	555	2,2					PB000224.01.0	PC000396.02.0	PY02953	methyltransferase, putative
PF14_0350	245	536	2,2			yes		PB000637.00.0	PC000449.04.0	NA	Acyl-CoA N-acyltransferase protein, putative
PF10_0120	464	971	2,1					PB000269.03.0	PC301404.00.0	PY03477	ubiquinol-cytochrome c reductase complex subunit, putative
PFC0995c	397	844	2,1			yes		PB001152.01.0	PC301445.00.0	PY01256	acyl CoA:diacylglycerol acyltransferase, putative

PF11_0174	549	1154	2,1		yes			PB000856.03.0	PC000818.02.0	PY05365	cathepsin C, homolog
**nucleic metabolism**											
PFL1005c	161	1731	10,8					PB000771.00.0	PC302534.00.0	PY04907	chromodomain protein
PFC0920w	308	3107	10,1			yes	yes	PB001096.02.0	PC000384.04.0	PY00436	histone H2A variant, putative
PFF0860c	439	4099	9,3	yes		yes		PB300144.00.0	PC000638.00.0	PY05076	histone h2a
PF14_0695	148	1101	7,4			yes		PB000837.02.0	PC300200.00.0	PY04295	DNA-directed RNA polymerase alpha subunit truncated putative
PFL0635c	195	1379	7,1			yes		PB000190.02.0	PC000050.03.0	PY04922	bromodomain protein putative
PF11_0091	707	4187	5,9			yes		PB001077.01.0	PC301184.00.0	PY07039	transcription factor with AP2 domain(s), putative
PF14_0633	471	2647	5,6					PB000752.01.0	PC000659.02.0	PY00247	transcription factor with AP2 domain(s), putative
PFC0465c	797	4444	5,6					PB001198.00.0	PC000763.01.0	PY05315	Pre-mRNA splicing factor, putative
PF10_0063	164	903	5,5				yes	PB000878.02.0	PC000206.00.0	NA	DNA/ RNA-binding protein, putative
PFD0825c	321	1705	5,3			yes		PB000416.02.0	PC001161.02.0	PY04369	RNA-binding protein of pumilio mpt5 family putative
PFF0865w	702	3368	4,8				yes	NA	NA	PY05073	histone h3
PF11_0061	442	2009	4,5				yes	NA	NA	NA	histone H4, putative
PF07_0054	239	1000	4,2	yes			yes	PB001306.02.0	PC000354.03.0	PY02616	histone h2b putative
PFI0820c	277	1130	4,1					PB000805.02.0	PC000519.00.0	PY06963	RNA-binding protein putative
PFL2140c	650	2555	3,9	yes		yes	yes	PB000586.02.0	PC001140.02.0	PY03828	ADP-ribosylation factor GTPase-activating protein
PF10_0214	264	995	3,8			yes		PB000204.01.0	PC000285.00.0	PY04813	RNA binding protein, putative
PF11_0319	667	2513	3,8			yes		PB300007.00.0	PC000548.01.0	PY01166	mitochondrial rpoD precursor, putative
PFD0985w	349	1297	3,7			yes		PB000863.01.0	PC000577.00.0	PY06328	transcription factor with AP2 domain(s), putative
PFF0510w	834	3001	3,6					NA	NA	PY00496	histone H3 putative
PFF1100c	765	2689	3,5			yes		PB000790.01.0	PC000091.02.0	PY02180	transcription factor with AP2 domain(s), putative
PF13_0142	340	1184	3,5					PB000186.00.0	PC000526.03.0	PY00919	Lsm6 homologue, putative
PF08_0096	320	1105	3,5					PB000057.03.0	PC000292.01.0	PY02224	RNA helicase putative
PF14_0413	864	2767	3,2			yes		PB000880.03.0	PC000337.02.0	PY01911	CAF1 family ribonuclease, putative
PF08_0074	162	508	3,1				yes	PB000862.00.0	PC000311.03.0	PY07825	DNA/RNA-binding protein Alba, putative
PFE0980c	990	3057	3,1			yes	yes	PB000509.03.0	PC000750.04.0	PY04723	CCR4
MAL8P1.104	209	640	3,1				yes	PB001232.00.0	PC000895.01.0	PY01168	CAF1 family ribonuclease, putative
MAL13P1.338	167	505	3,0					PB000127.02.0	PC000638.04.0	PY00080	u1 small nuclear ribonucleoprotein putative
PF11_0264	363	1090	3,0				yes	PB000901.00.0	PC000152.03.0	PY06559	DNA-dependent RNA polymerase
PF14_0436	225	676	3,0			yes		PB000433.03.0	NA	PY01902	helicase truncated putative
PF11_0062	1038	2954	2,8				yes	PB001051.00.0	PC302253.00.0	NA	histone H2B
PF10_0232	450	1265	2,8			yes	yes	PB001011.00.0	PC000696.01.0	PY02297	Chromodomain-helicase-DNA-binding protein 1 homolog, putative
MAL8P1.70	144	393	2,7					PB001600.02.0	NA	PY03958	Zinc finger C-x8-C-x5-C-x3-H type, putative
PFL0145c	400	1077	2,7					PB000540.03.0	PC000607.03.0	PY05184	high mobility group protein, PfHMGB1
PF14_0150	188	500	2,7					PB000985.02.0	PC000134.02.0	PY04455	RNA polymerase small subunit putative
PF14_0433	266	706	2,7	yes				PB001120.01.0	PC000347.02.0	PY05556	erythrocyte membrane-like protein
PF07_0126	715	1872	2,6			yes	yes	PB000422.00.0	PC000723.01.0	PY01583	transcription factor with AP2 domain(s), putative
PF11_0053	1139	2971	2,6					PB000313.01.0	PC000596.01.0	PY02376	PfSNF2L
PFE0500c	1245	3150	2,5				yes	NA	NA	PY04034	RRM and KH domain protein
PF11_0163	173	438	2,5					PB000983.00.0	PC001239.02.0	PY00782	transcription factor with AP2 domain(s), putative
PFF1185w	270	682	2,5			yes		PB000309.00.0	PC001116.02.0	PY05642	iswi protein homologue
PF10_0083	291	724	2,5	yes				PB000525.02.0	NA	PY05712	S1 (Kaiser et al 2004) / Zinc finger C-x8-C-x5-C-x3-H type, putative
PF14_0489	272	665	2,4			yes	yes	PB000549.02.0	PC000648.02.0	PY01626	histone deacetylase, putative
PFF0200c	209	510	2,4					PB300561.00.0	PC000219.00.0	PY05930	transcription factor with AP2 domain(s), putative
PFI0210c	818	1994	2,4		yes	yes		PB000791.02.0	PC000123.03.0	PY01148	CCAAT-box DNA binding protein, putative
PFI0165c	434	1055	2,4			yes		PB001045.00.0	PC300325.00.0	PY01108	DEAD DEAH box helicase putative / protein recongnized by volunteers immunized by irradied *P.falciparum* sporozoite (Doolan et al 2003)
PF14_0278	1992	4745	2,4			yes		PB000339.03.0	PC000889.02.0	PY01271	ATP-dependent DNA helicase putative
PFC0440c	1040	2466	2,4			yes	yes	NA	NA	NA	DEAD box helicase, putative
PFD0565c	243	571	2,3			yes	yes	PB000982.01.0	PC300524.00.0	PY04868	conserved apicomplexan protein|DEAD box ATP-dependent RNA helicase, putative
PF08_0055	244	568	2,3	yes		yes		NA	PC000115.01.0	PY03839	u3 small nucleolar ribonucleoprotein protein putative
PF14_0183	418	972	2,3			yes		PB000865.03.0	PC000372.05.0	PY06632	RNA helicase putative
PF10_0309	385	883	2,3			yes		NA	NA	NA	DEAD DEAH box helicase, putative
MAL8P1.40	330	757	2,3			yes		PB000007.00.0	PC000004.04.0	PY02680	RNA-binding protein putative
PF07_0027	307	692	2,3					PB300575.00.0	PC000255.04.0	PY00134	DNA-directed RNA polymerase 2 8.2 kDa polypeptide putative
PFF1425w	552	1237	2,2					NA	PC301509.00.0	PY04169	RNA binding protein, putative
PF11_0250	13879	30709	2,2				yes	PB000394.00.0	PC000674.04.0	PY03136	high mobility group-like protein NHP2 putative
MAL7P1.21	511	1115	2,2					PB000792.01.0	PC000419.02.0	PY03235	origin recognition complex subunit putative
MAL13P1.242	169	362	2,1					PB001275.00.0	NA	PY02626	step II splicing factor, putative
PFC0130c	150	318	2,1					PB000610.01.0	PC000347.03.0	PY03963	RNA binding protein, conserved
PFL0330c	171	360	2,1			yes	yes	PB001119.03.0	PC301400.00.0	PY01847	DNA-directed RNA polymerase III subunit putative
MAL8P1.72	193	406	2,1				yes	PB001601.02.0	PC000449.02.0	PY07077	high mobility group protein putative, PfHMGB2
PF10_0047	389	819	2,1					PB001621.02.0	PC000074.04.0	PY05537	RNA binding protein, putative
PFL0465c	497	1033	2,1				yes	PB000307.00.0	PC000051.01.0	PY03615	Zinc finger transcription factor (krox1)
PFF0350w	370	769	2,1			yes		PB300939.00.0	PC300643.00.0	PY00650	MYND finger protein
PFI0455w	2182	4505	2,1			yes		PB000392.00.0	PC000757.01.0	PY03131	exoribonuclease putative
PF13_0152	251	514	2,0			yes		PB000839.01.0	PC000625.00.0	PY01554	transcriptional regulatory protein sir2 homologue putative
PFE0090w	641	1310	2,0			yes		PB001058.03.0	PC000005.05.0	PY03920	chromosome assembly factor 1, CAF-1

MAL13P1.32	3157	6319	2,0			yes	yes	PB000953.02.0	PC000879.02.0	PY02591	MORN repeat containing protein
**protein metabolism**											
PFL2095w	520	6291	12,1					PB000550.03.0	NA	PY01209	Translation initiation factor SUI1 putative
MAL7P1.93	196	1602	8,2			yes		PB000551.01.0	PC000600.03.0	PY05271	mitochondrial ribosomal protein S8 putative
PF11_0313	172	947	5,5					PB000391.01.0	PC000236.04.0	PY00659	60S ribosomal protein P0
PF11_0212	234	1071	4,6		yes	yes		PB000097.03.0	PC000693.01.0	PY00891	tRNA nucleotidyltransferase, putative
PF14_0231	615	2790	4,5	yes		yes		PB000126.01.0	PC000974.01.0	PY06460	60S ribosomal protein L7-3, putative
PFF1395c	226	1014	4,5			yes	yes	PB000738.01.0	PC000003.04.0	PY03547	glutamyl-tRNA(Gln) amidotransferase subunit B putative
PF14_0448	158	705	4,5			yes	yes	PB001359.02.0	PC000201.02.0	PY06704	40S ribosomal protein S2, putative
PFC1000w	2962	11597	3,9			yes		PB001153.01.0	PC001221.02.0	PY01257	regulator of initiation factor 2 (eIF2)
PFL0500w	387	1391	3,6			yes		NA	PC000839.03.0	PY03485	mitochondrial ribosomal protein L1-2 precursor, putative
PFC1020c	194	638	3,3					PB001643.02.0	PC000155.05.0	PY02291	40S ribosomal protein S3A, putative
PFL0570c	400	1213	3,0					PB000926.03.0	PC000392.04.0	PY01223	mitochondrial ribosomal protein S18 precursor, putative
PF10_0183	152	452	3,0			yes		PB001219.02.0	PC000654.02.0	PY04040	eukaryotic translation initiation factor subunit eIF2A, putative
PF14_0585	202	583	2,9					PB000756.01.0	PC000029.04.0	PY04142	40S ribosomal protein S28e, putative
PF13_0213	213	598	2,8					PB000686.02.0	PC302562.00.0	PY02722	60S ribosomal protein L6-2, putative
PFB0175c	534	1402	2,6			yes		PB000405.03.0	PC300148.00.0	PY01531	nucleolar preribosomal assembly protein, putative
PF10_0264	156	402	2,6			yes		PB000415.00.0	PC000391.02.0	PY06059	40S ribosomal protein S2B, putative
PFC0870w	7271	18069	2,5					PB000958.03.0	PC000580.02.0	NA	elongation factor 1 (EF-1), putative
PFF1305w	1004	2455	2,4					PB000062.03.0	PC001125.02.0	PY04644	mitochondrial ribosomal protein L46 precursor, putative
PFI1510w	205	497	2,4			yes		PB300365.00.0	PC301601.00.0	PY05559	Nucleolar protein Nop52, putative
PFL1590c	261	626	2,4			yes		PB001145.00.0	PC000547.00.0	PY04706	elongation factor g putative
PF14_0627	167	386	2,3			yes	yes	PB000822.02.0	PC301233.00.0	NA	40S ribosomal protein S3, putative
PFC0400w	220	499	2,3			yes	yes	PB000523.01.0	PC000329.02.0	PY02905	60S Acidic ribosomal protein P2, putative
PF13_0268	248	561	2,3					PB001521.02.0	PC001057.02.0	PY00934	60S ribosomal protein L17, putative
PF08_0011	853	1859	2,2		yes	yes	yes	PB000524.03.0	PC000269.05.0	PY05778	leucine -- tRNA ligase
PF10_0313	1516	3238	2,1			yes	yes	PB000814.01.0	PC000473.03.0	PY04560	mitochondrial preribosomal assembly protein rimM precursor, putative
MAL13P1.283	195	411	2,1			yes	yes	PB001077.00.0	PC001405.02.0	PY02937	TCP1 chaperonin delta subunit putative
PFL0335c	544	1096	2,0					PB000468.02.0	PC301070.00.0	PY07150	eukaryotic translation initiation factor 5 putative
**ubiquitin signalling pathway**											
PF13_0346	918	7010	7,6	yes				PB000533.00.0	PC000735.00.0	NA	60S ribosomal protein L40/UBI, putative
PFL0585w	794	5982	7,5	yes				NA	NA	PY03971	PfpUB Plasmodium falciparum polyubiquitin
PFI1545c	174	475	2,7			yes		PB000332.00.0	PC000230.01.0	PY03212	proteosome precursor putative
PF11_0329	335	891	2,7					PB000689.01.0	PC000851.00.0	PY03631	ubiquitin-like protein
PFL0190w	211	497	2,4			yes		PB000336.03.0	PC000554.00.0	PY03025	ubiquitin-conjugating enzyme e2 putative
PF10_0174	318	685	2,2					PB001167.00.0	PC001032.02.0	PY02643	26s proteasome subunit p55 putative
PF14_0373	146	308	2,1			yes		PB000018.02.0	PC000569.04.0	PY05634	ubiquinol cytochrome c oxidoreductase putative
MAL8P1.122	181	367	2,0			yes		PB000783.03.0	PC000216.03.0	PY01891	Ubiquitin regulatory protein, putative
PFC0745c	183	368	2,0	yes		yes		PB000672.03.0	PC000739.02.0	PY00152	proteasome component C8 putative
**protease**											
PFI0580c	1132	11153	9,9		yes		yes	PB000502.02.0	PC000277.02.0	NA	Falstatin, putative
PF08_0108	358	2811	7,9		yes	yes	yes	PB001294.02.0	PC000654.04.0	PY06692	pepsinogen putative
PFI0135c	304	1913	6,3		yes		yes	NA	NA	NA	papain family cysteine protease, putative|serine repeat antigen 9 (SERA-9)
PF11_0150	693	2809	4,1			yes		PB000352.00.0	PC000773.00.0	PY00729	PfROM1 (Baker et al 2006)
PF14_0110	210	818	3,9			yes		PB000753.00.0	NA	PY00165	rhomboid protease, putative
PFE0340c	330	992	3,0			yes		PB001432.02.0	PC000063.05.0	PY04351	PfROM4 (Baker et al 2006)
PF11_0162	341	759	2,2			yes		NA	NA	PY00783	falcipain-3
PFE0355c	193	390	2,0			yes		PB000701.02.0	NA	NA	serine protease belonging to subtilisin family, putative
**signalling /regulation**											
PF11_0139	304	2680	8,8			yes		PB000082.01.0	PC000676.00.0	PY05564	protein tyrosine phosphatase putative
PFE0455w	1398	7361	5,3					PB000365.01.0	PC001010.02.0	PY03257	phosphatase 1 regulatory subunit, putative
PFE0610c	349	1767	5,1					PB000948.00.0	PC000493.01.0	NA	CDK-activating kinase assembly factor, putative
MAL8P1.69	507	2327	4,6				yes	PB000900.00.0	PC000316.05.0	PY01841	14-3-3 protein
PFL2325c	704	2978	4,2			yes		PB000244.02.0	PC000413.04.0	PY00378	P. falciparum homologue of Drosophila nmda1 protein putative
PF11_0225	874	3384	3,9			yes		PB000693.01.0	NA	PY06050	PfGCN20
PFA0380w	278	995	3,6			yes	yes	PB001255.02.0	PC001368.02.0	PY06752	serine threonine protein kinase putative
PFD0975w	340	1025	3,0			yes		PB000429.00.0	PC000025.01.0	PY06287	Atypical protein kinase, RIO family, putative
PF14_0408	303	908	3,0			yes		PB000491.01.0	PC000532.01.0	PY00095	Ser Thr protein kinase putative
PF14_0734	641	1882	2,9			yes		NA	NA	NA	Serine/Threonine protein kinase, FIKK family
PFB0665w	248	675	2,7			yes		PB000061.02.0	PC301925.00.0	PY06391	serine/threonine protein kinase, putative
PF11_0464	3490	9431	2,7		yes	yes	yes	PB000477.02.0	PC000083.05.0	PY00717	Serine/Threonine protein kinase, putative
PFD1180w	929	2408	2,6			yes		NA	NA	NA	trophozoite antigen r45-like protein,truncated
PF11_0396	381	948	2,5			yes		PB000103.02.0	PC000065.00.0	PY06845	Protein phosphatase 2C
PFL2250c	146	362	2,5					PB000522.03.0	PC000605.04.0	PY00403	rac-beta serine/threonine protein kinase, PfPKB
PF13_0166	667	1640	2,5			yes		PB106157.00.0	PC103301.00.0	PY02207	protein kinase putative
PF14_0614	1202	2732	2,3		yes	yes	yes	PB000092.02.0	PC000578.04.0	PY04404	serine/threonine protein phosphatase, putative
MAL13P1.275	241	503	2,1					PB001065.03.0	PC000193.04.0	PY04698	NLI interacting factor-like phosphatase, putative
PFI1280c	712	1474	2,1			yes	yes	PB001259.00.0	PC000888.01.0	PY00761	protein kinase putative

PF10_0311	176	359	2,0					NA	NA	NA	protein phosphatase inhibitor, putative
PF13_0011	277	562	2,0					NA	NA	NA	plasmodium falciparum gamete antigen 27 25
PF07_0024	260	527	2,0					PB001142.02.0	PC001299.02.0	PY03237	inositol phosphatase, putative
**transportation**											
PFI1590c	273	2430	8,9			yes		PB000770.03.0	PC000292.05.0	PY04917	CAS/CSE protein, putative
PFA0245w	114	911	8,0			yes	yes	PB001038.01.0	PC000510.04.0	PY07333	novel putative transporter, PfNPT
PFB0275w	375	2747	7,3			yes		PB001031.03.0	PC000807.04.0	PY05039	metabolite/drug transporter|Major Facilitator Superfamily, putative
PFE0775c	1269	6073	4,8			yes		PB300327.00.0	PC000864.01.0	PY02168	amino acid transporter
PFF1345w	266	1196	4,5			yes		PB000458.03.0	PC000325.02.0	PY02706	transportin
PFD0745c	331	1363	4,1			yes		NA	PC000138.04.0	PY04027	nonclathrin coat protein zeta2-cop-related protein, putative
PF11_0461	658	2443	3,7			yes		PB300286.00.0	PC000978.01.0	PY00721	PfRab6, GTPase
PFC0725c	448	1397	3,1			yes	yes	PB000183.01.0	PC001071.02.0	PY06388	formate-nitrate transporter, putative
PF14_0679	368	1075	2,9			yes		PB000598.03.0	PC000144.02.0	PY07224	inorganic anion exchanger, inorganic anion antiporter
PF14_0654	367	1029	2,8			yes		NA	NA	NA	aminophospholipid transporter activity
PF11_0310	195	524	2,7			yes		PB000390.01.0	PC301882.00.0	PY02129	transporter putative
PF13_0124	161	417	2,6			yes		PB000044.02.0	PC302473.00.0	PY06857	SFT2=like protein
PFA0375c	254	656	2,6			yes	yes	PB001237.02.0	PC000233.03.0	PY03953	lipidsterol:H+ symporter
PF13_0174	179	422	2,4			yes		PB000322.02.0	PC000246.01.0	PY05729	P. falciparum homologue of human mbp-1 interacting protein-2a; putative
MAL8P1.51	356	820	2,3			yes		NA	NA	NA	protein-transport protein sec61 beta 1 subunit, putative
PFE1455w	4195	9641	2,3			yes		NA	NA	NA	sugar transporter, putative
PF13_0358	146	337	2,3					PB001078.03.0	PC000028.03.0	PY05705	mitochondrial import inner membrane translocase, putative
PFA0310c	191	437	2,3			yes		PB000675.00.0	PC301192.00.0	PY05776	calcium-transporting ATPase
PF10_0366	1507	3435	2,3			yes	yes	PB001072.01.0	PC000019.01.0	PY03882	ADPATP transporter on adenylate translocase
PF13_0324	348	762	2,2					PB001372.02.0	PC300976.00.0	PY01094	Sec24 subunit, putative
PF11_0258	622	1354	2,2	yes				PB300486.00.0	PC000284.03.0	PY00817	co-chaperone GrpE, putative
PFL0785c	244	518	2,1					PB001194.02.0	NA	PY05584	signal recognition particle 19 kd protein, putative
PFE1195w	321	677	2,1			yes	yes	PB001230.00.0	PC000072.05.0	PY00155	karyopherin beta
PF14_0221	733	1520	2,1	yes			yes	PB000271.02.0	PC000122.02.0	PY00019	GTPase of unknown function, putative
**cytoskeleton**											
PF11_0431	740	2573	3,5					PB000461.01.0	PC301320.00.0	PY03535	membrane skeletal protein putative
PFL2215w	405	1405	3,5			yes	yes	PB000323.01.0	NA	PY02240	actin
PFC0860w	498	1456	2,9			yes		PB000348.03.0	PC000825.01.0	PY00972	kinesin, putative
PFE0175c	356	994	2,8	yes		yes	yes	PB000366.02.0	PC000242.02.0	PY00345	unconventional myosin pfm-b
PF13_0233	456	1117	2,4			yes	yes	PB001069.00.0	PC001166.02.0	PY01232	myosin a
PF14_0222	545	1301	2,4				yes	PB000272.02.0	PC000123.02.0	PY00018	Ankyrin, putative
PF14_0202	175	406	2,3					PB000808.00.0	PC000364.02.0	NA	dynein-associated protein, putative
PFC0165w	450	1000	2,2					PB001240.02.0	PC000020.05.0	PY00501	spindle pole body protein, putative
PFI1080w	236	491	2,1			yes		PB001113.01.0	PC001267.02.0	PY00184	dynein intermediate chain 2, ciliary
**stress responce**											
PF08_0054	639	15606	24,4	yes		yes	yes	PB000817.02.0	PC000186.01.0	PY06158	heat shock 70 kDa protein
PF07_0029	304	5761	19,0	yes		yes	yes	PB300823.00.0	PC000221.01.0	PY00131	heat shock protein 86
PFL0565w	194	1383	7,1					PB000927.03.0	PC000656.04.0	PY01224	heat shock protein DNAJ homologue Pfj4
PFI0875w	1118	6610	5,9		yes	yes	yes	PB001177.00.0	PC300345.00.0	PY05001	Heat shock protein
PF11_0351	239	968	4,1			yes	yes	PB001074.01.0	PC001357.02.0	PY06981	heat shock protein hsp70 homologue
PF11_0175	194	717	3,7		yes	yes	yes	PB001190.00.0	PC000279.05.0	PY05364	heat shock protein 101 putative
PFE1605w	766	2504	3,3			yes		NA	NA	NA	protein with DNAJ domain /PHIST domain protein
PFC0360w	122	363	3,0					PB000835.01.0	PC000094.03.0	NA	Activator of Hsp90 ATPase homolog 1-like protein, putative
PF11_0099	287	807	2,8		yes	yes		PB001000.01.0	PC301594.00.0	PY07174	heat shock protein DnaJ homologue Pfj2
PFF1130c	294	704	2,4		yes		yes	PB000741.00.0	PC000388.01.0	PY04892	superoxide dismutase, PfSOD2
PF14_0137	970	2307	2,4			yes		PB000205.01.0	PC000928.01.0	PY05607	protein with DNAJ domain
PF10_0378	474	967	2,0			yes		NA	NA	NA	RESA-like protein with PHIST and DnaJ domains
PF14_0013	677	1372	2,0			yes		NA	NA	NA	DnaJ protein, putative
PF11_0273	145	288	2,0			yes		NA	PC300089.00.0	NA	DnaJ protein, putative
PF14_0700	196	393	2,0					PB000935.01.0	PC000353.05.0	PY03688	DnaJ protein, putative
**blood stage surface proteins**											
PFF1555w	288	1642	5,7		yes	yes		NA	NA	NA	rifin
PF13_0193	516	2890	5,6		yes			NA	NA	NA	MSP7-like protein
PF14_0201	212	964	4,5		yes	yes	yes	PB000807.00.0	PC000379.04.0	PY05715	surface protein, putative, Pf113
PFF0620c	200	888	4,4		yes	yes		PB000527.00.0	PC000586.03.0	PY03099	Pfs45-48 related protein, putative
PFD0100c	340	1110	3,3				yes	NA	NA	NA	surface-associated interspersed gene 4.1, (SURFIN4.1)
MAL7P1.12	257	711	2,8			yes		PB000800.03.0	PC000194.03.0	PY00683	erythrocyte membrane-associated antigen
MAL7P1.12	224	612	2,7			yes		PB000800.03.0	PC000194.03.0	PY00683	erythrocyte membrane-associated antigen
PFB0935w	297	782	2,6		yes	yes		NA	NA	NA	cytoadherence linked asexual protein 2
PFF0020c	248	627	2,5			yes		NA	NA	NA	Plasmodium falciparum var-like protein
MAL7P1.1	145	367	2,5			yes		NA	NA	NA	erythrocyte membrane protein 1 (PfEMP1)
PF10_0003	290	721	2,5		yes	yes		NA	NA	NA	rifin
PFF1540w	287	674	2,3			yes		NA	NA	NA	RIF pseudogene
PFC1070c	557	1233	2,2					NA	NA	NA	VARC pseudogene

MAL7P1.7	617	1327	2,2			yes		NA	NA	NA	RESA-like protein/ PHIST domain protein
PFL0935c	285	602	2,1			yes		NA	NA	NA	erythrocyte membrane protein 1 (PfEMP1)
PFA0765c	910	1895	2,1			yes		NA	NA	NA	erythrocyte membrane protein 1 (PfEMP1)
**parasitophorous vacuole proteins**											
MAL8P1.6	467	13807	29,6			yes		NA	NA	NA	early transcribed membrane protein 8, ETRAMP 8 (Spielmann et al 2003)
PFL0065w	395	11094	28,1		yes	yes		NA	NA	NA	LSAP-1
PF14_0729	308	5102	16,6			yes		NA	NA	NA	etramp14.2 (Spielmann et al 2003)
PFB0105c	611	8550	14,0			yes		NA	NA	NA	LSAP-2/ PHIST domain protein
PF10_0164	714	4459	6,2		yes	yes		NA	NA	NA	etramp10.3 (Spielmann et al 2003)/ UIS4 (Mueller et al 2005)
PF14_0678	967	5684	5,9			yes		PB000390.00.0	PC000056.05.0	PY05892	exported protein 2 (Fisher et al 1998)
PF13_0012	205	883	4,3	yes	yes	yes		PB000892.03.0	PC000203.01.0	PY03011	etramp13 (Splieman et al 2003)/UIS3 (Mikolajczak et al 2007)
PFD1120c	245	737	3,0		yes	yes		NA	NA	NA	etramp4 (Spielmann et al 2003)
PF11_0224	344	936	2,7		yes	yes		PB000484.01.0	PC000214.05.0	PY04421	circumsporozoite-related antigen / exported protein 1 (Doolan et al 1996)
PF10_0344	277	586	2,1		yes		yes	NA	NA	NA	glutamate-rich protein /pfGLURP (Borre et al 1991)
**hypothetical proteins**											
PF08_0119	1236	20913	16,9					PB000508.03.0	PC000587.00.0	NA	hypothetical protein, conserved
PF10_0190	153	1650	10,8					NA	NA	NA	hypothetical protein
PF14_0046	250	2506	10,0		yes			NA	NA	NA	hypothetical conserved in Plasmodium species
PF11_0340	319	2587	8,1					NA	NA	NA	hypothetical protein
PF13_0203	925	7484	8,1		yes			PB104029.00.0	PC405853.00.0	NA	hypothetical protein
PFE0990w	197	1244	6,3					PB000706.03.0	NA	PY00700	hypothetical protein, conserved
PF14_0336	562	3000	5,3					NA	NA	PY05583	hypothetical protein
PFC0615w	446	2358	5,3					PB000077.03.0	NA	PY07280	hypothetical protein
PF11_0499	72624	373683	5,1					NA	NA	NA	hypothetical protein
PF08_0082	201	1023	5,1				yes	PB000301.02.0	NA	PY05997	hypothetical protein
PF08_0083	224	1031	4,6					PB000460.03.0	NA	PY03189	hypothetical protein
PFI0325c	242	1114	4,6					PB300478.00.0	PC000617.00.0	NA	hypothetical protein
PF13_0288	674	2875	4,3	yes				PB001076.02.0	PC000662.00.0	NA	hypothetical protein
PFA0360c	300	1267	4,2		yes			NA	NA	NA	hypothetical protein
PF10_0060	933	3911	4,2		yes			PB000475.03.0	PC001364.02.0	PY06601	hypothetical protein
PF14_0080	344	1433	4,2					NA	NA	NA	hypothetical protein
PFE0115c	312	1291	4,1				yes	NA	PC000724.04.0	NA	hypothetical protein
PF14_0621	258	1044	4,0					PB000781.03.0	PC000515.01.0	PY01369	hypothetical protein
PF11_0497	140465	561120	4,0					NA	NA	NA	hypothetical protein
PFL1770c	309	1220	3,9				yes	PB000156.01.0	PC000341.01.0	PY05543	hypothetical protein
MAL13P1.125	252	975	3,9					NA	NA	NA	hypothetical protein
PFA0135w	157	601	3,8		yes			NA	NA	NA	Merozoite-associated tryptophan-rich antigen, putative
PFE1500c	811	3065	3,8					NA	PC100686.00.0	NA	hypothetical protein
PFE1100w	154	580	3,8					NA	NA	NA	hypothetical protein
PF10_0357	6572	24359	3,7					NA	NA	NA	hypothetical protein
MAL7P1.160	413	1519	3,7					NA	PC000211.01.0	NA	hypothetical protein
PF13_0167	352	1269	3,6					PB404030.00.0	NA	PY00481	hypothetical protein
PFI1270w	275	984	3,6		yes			PB000110.02.0	NA	PY00763	hypothetical protein
PF13_0058	1413	5034	3,6				yes	PB300785.00.0	PC000772.00.0	PY02798	hypothetical protein
PFF0525w	168	600	3,6					NA	NA	NA	hypothetical protein
PF11_0418	190	675	3,6					PB000851.03.0	PC102167.00.0	NA	hypothetical protein
MAL7P1.125	347	1218	3,5					PB000811.01.0	PC000195.01.0	PY02049	hypothetical protein
PF11_0494	17658	61213	3,5					NA	PC300798.00.0	NA	hypothetical protein
PF11_0253	194	670	3,5					PB301523.00.0	PC000624.02.0	NA	hypothetical protein
PFL1765c	240	808	3,4					PB000388.00.0	NA	PY06122	hypothetical protein
PFE0615w	295	986	3,3					NA	PC106709.00.0	PY03183	hypothetical protein
MAL13P1.182	292	954	3,3					PB001042.01.0	PC000515.03.0	PY01503	hypothetical protein, conserved
MAL8P1.8	182	592	3,3					PB000506.01.0	PC000739.04.0	PY06580	hypothetical protein
PF14_0344	4602	14792	3,2		yes		yes	PB000730.02.0	PC000742.04.0	PY02301	hypothetical protein
PFF1310c	158	500	3,2					NA	PC001126.02.0	NA	hypothetical protein
MAL13P1.24	170	535	3,1		yes			PB000071.01.0	PC000080.03.0	PY07311	hypothetical protein
PF10_0170	340	1047	3,1				yes	NA	PC000204.05.0	PY02639	hypothetical protein
PF11_0322	310	952	3,1				yes	NA	PC300184.00.0	PY06085	hypothetical protein
PF13_0161	1906	5813	3,0					PB000553.03.0	PC000775.02.0	PY00261	hypothetical protein
PF14_0089	614	1862	3,0					NA	NA	PY03352	hypothetical protein
PF11_0302	306	915	3,0		yes			NA	NA	NA	hypothetical protein
PF14_0461	6158	18306	3,0				yes	NA	PC000800.03.0	NA	hypothetical protein
PF07_0044	408	1186	2,9					PB000240.00.0	PC103612.00.0	PY04862	hypothetical protein
PF14_0044	219	635	2,9		yes			PB000264.00.0	PC300261.00.0	PY06764	S11 (Kaiser et al 2004)
PFL0845w	407	1177	2,9					PB000694.01.0	PC000170.02.0	PY04727	hypothetical protein
PF14_0684	421	1216	2,9					NA	NA	NA	hypothetical protein
PF14_0093	1540	4442	2,9				yes	NA	NA	NA	hypothetical protein
PF14_0490	2908	8351	2,9					PB001087.00.0	PC000649.02.0	PY01625	hypothetical protein

PF14_0140	243	681	2,8					PB000384.00.0	PC000163.04.0	PY03289	hypothetical protein
PFI1075w	581	1616	2,8					NA	PC103190.00.0	NA	hypothetical protein
PF11_0526	2007	5569	2,8					NA	PC000254.03.0	NA	hypothetical protein
PF14_0707	696	1903	2,7					PB000387.00.0	NA	PY05083	hypothetical protein
PF11_0118	397	1079	2,7					PB000660.02.0	PC001396.02.0	PY02705	hypothetical protein
PFL0795c	368	1000	2,7		yes			PB000248.02.0	PC000315.05.0	PY00179	pfmvd, male development gene
PFD0785c	568	1533	2,7					PB300755.00.0	PC000236.02.0	NA	hypothetical protein
PF13_0200	723	1942	2,7					PB000375.03.0	PC000405.02.0	PY03051	hypothetical protein
PF11_0166	230	618	2,7					NA	NA	NA	hypothetical protein
PF11_0473	233	625	2,7					PB000941.03.0	PC000759.01.0	PY03510	hypothetical protein
PFL1175w	455	1216	2,7					PB000904.03.0	PC000213.00.0	PY05853	hypothetical protein
PFC0215c	188	497	2,6		yes			NA	NA	NA	hypothetical protein
PF10_0231	464	1215	2,6					PB300797.00.0	PC000436.00.0	PY02296	hypothetical protein
PFL2025w	200	522	2,6					PB000043.03.0	PC000282.02.0	PY01489	hypothetical protein
PF08_0118	2220	5769	2,6					PB000507.03.0	PC300547.00.0	PY05558	hypothetical protein
PFI1235w	258	670	2,6	yes			yes	PB000907.02.0	PC000856.01.0	PY00928	hypothetical protein
PF10_0204	176	455	2,6					PB001641.02.0	PC000478.00.0	PY03164	hypothetical protein
PF14_0665	546	1408	2,6					PB000495.02.0	PC000205.05.0	PY01696	hypothetical protein
PF11_0406	320	820	2,6					NA	NA	NA	hypothetical protein
PFC0555c	898	2290	2,6					PB000259.00.0	PC000760.00.0	PY03661	hypothetical protein
PF14_0685	196	491	2,5					NA	NA	NA	hypothetical protein
PF14_0715	316	786	2,5					PB000784.03.0	PC000561.04.0	PY00266	hypothetical protein
PF13_0160	155	379	2,4					PB000554.03.0	PC301602.00.0	NA	hypothetical protein
PFA0305c	258	631	2,4					NA	PC000804.03.0	PY05775	hypothetical protein
PFA0210c	235	571	2,4		yes			PB300987.00.0	NA	PY06307	hypothetical protein
MAL13P1.331	223	542	2,4					NA	NA	PY05033	hypothetical protein conserved
PFD0900w	282	683	2,4					NA	PC000330.01.0	PY06816	hypothetical protein
PF10_0342	747	1799	2,4		yes			NA	NA	NA	hypothetical protein
PF11_0415	470	1124	2,4					PB000010.01.0	PC000423.00.0	PY00530	hypothetical protein
PF14_0713	216	513	2,4					PB000964.02.0	PC301612.00.0	PY02637	hypothetical protein
PFF1230c	501	1185	2,4		yes			NA	PC000158.00.0	PY02327	hypothetical protein
PFD0010w	252	594	2,4					NA	NA	NA	unknown
PF11_0320	12380	28928	2,3					NA	NA	NA	hypothetical protein
PF13_0204	235	550	2,3					PB000571.02.0	PC301210.00.0	PY06251	nuclear movement protein, putative
PF13_0191	775	1805	2,3		yes		yes	NA	NA	NA	hypothetical protein
PF13_0295	204	472	2,3					NA	NA	NA	hypothetical protein
PF11_0209	552	1271	2,3		yes			PB000141.01.0	NA	PY00888	hypothetical protein
PFB0775w	613	1412	2,3					NA	PC000481.00.0	PY04634	hypothetical protein
PF11_0033	165	380	2,3					NA	NA	NA	hypothetical protein
PF07_0120	337	759	2,3					PB000175.01.0	PC000650.02.0	PY01476	hypothetical protein
PFF0165c	224	504	2,3					PB001170.02.0	PC000690.00.0	PY02581	hypothetical protein
PFC0910w	200	448	2,2					PB300241.00.0	PC000219.04.0	PY05269	hypothetical protein
PFE0780w	999	2234	2,2					PB000695.03.0	PC000751.01.0	PY02013	hypothetical protein
MAL13P1.180	217	485	2,2	yes				PB402792.00.0	PC102239.00.0	NA	hypothetical protein
PF10_0180	1445	3205	2,2	yes				PB000170.02.0	PC301007.00.0	PY02493	hypothetical protein
PF11_0365	413	914	2,2					PB001098.02.0	PC300229.00.0	PY03003	hypothetical protein
PF11_0501	38485	85105	2,2					NA	NA	NA	hypothetical protein
PFE1425c	145	322	2,2					NA	NA	PY00692	hypothetical protein
PF14_0390	1188	2615	2,2					NA	NA	PY05640	hypothetical protein
PFF0840w	212	467	2,2					PB000604.01.0	PC302191.00.0	PY05077	hypothetical protein
PF11_0462	297	652	2,2					NA	PC000709.02.0	NA	hypothetical protein
PF14_0426	209	455	2,2					PB107642.00.0	NA	NA	hypothetical protein
PFI0715w	198	430	2,2					PB000461.03.0	NA	PY06377	Zinc binding protein, putative
PFL0105w	633	1360	2,1					NA	NA	NA	hypothetical protein
PF14_0176	256	549	2,1				yes	PB000252.00.0	PC106247.00.0	PY01646	hypothetical protein
PFD0920w	312	667	2,1				yes	NA	PC300097.00.0	NA	hypothetical protein
PF11_0402	366	776	2,1					PB000321.01.0	PC000753.01.0	PY05866	hypothetical protein
MAL8P1.95	477	1010	2,1	yes			yes	PB000840.00.0	PC302359.00.0	PY00457	hypothetical protein
PF11_0160	189	397	2,1				yes	PB000981.00.0	PC000782.00.0	PY00784	hypothetical protein
PF10_0296	437	917	2,1					NA	PC000130.02.0	PY04570	hypothetical protein
PF14_0236	363	762	2,1				yes	NA	NA	NA	hypothetical protein
PF14_0339	528	1102	2,1					PB300666.00.0	PC301098.00.0	PY00616	hypothetical protein
PF11_0289	2216	4620	2,1					PB000435.00.0	PC000031.02.0	PY03552	hypothetical protein
PF13_0194	179	374	2,1		yes			NA	NA	NA	hypothetical protein
PF10_0234	408	842	2,1				yes	PB000312.02.0	NA	PY01460	hypothetical protein
PF13_0339	498	1025	2,1					PB000361.02.0	PC000252.05.0	PY00836	hypothetical protein
PFC0690c	305	627	2,1	yes			yes	PB000588.01.0	PC000431.02.0	PY05938	hypothetical protein
PF14_0238	24997	51289	2,1					NA	NA	NA	hypothetical protein
PFE0315c	183	375	2,0		yes			NA	NA	NA	hypothetical protein

PF14_0219	320	654	2,0					NA	NA	NA	hypothetical protein
PFL0910c	254	518	2,0					NA	PC000648.00.0	NA	hypothetical protein
PFI0915w	2539	5164	2,0					NA	NA	NA	hypothetical protein
PF14_0427	200	406	2,0					PB000454.03.0	PC302439.00.0	PY03707	hypothetical protein
PF10_0156	241	489	2,0					PB000457.03.0	PC000113.01.0	PY06645	hypothetical protein
PFI0675w	362	732	2,0					NA	NA	NA	hypothetical protein
PFD0480w	445	900	2,0		yes			PB300840.00.0	PC300699.00.0	PY04089	hypothetical protein
PF11_0378	209	420	2,0					PB000288.02.0	PC000776.03.0	PY05749	hypothetical protein
PFF0635w	267	536	2,0		yes			NA	NA	NA	hypothetical protein
PFF1295w	189	379	2,0					PB000191.03.0	PC000381.04.0	PY07532	hypothetical protein conserved
**hypothetical transmembrane proteins**											
PFL1430c	246	2405	9,8			yes		PB000347.03.0	PC000466.01.0	PY07335	hypothetical protein
PF14_0467	682	5504	8,1		yes	yes	yes	PB000658.00.0	PC301505.00.0	PY05966	hypothetical protein
PFF1315w	315	2240	7,1			yes		PB000068.01.0	PC000487.03.0	PY04642	hypothetical protein with ankyrin repeats
PFL0655w	592	4175	7,1		yes	yes		PB000768.01.0	PC302586.00.0	PY02015	hypothetical protein
PF11_0149	726	4648	6,4			yes		PB000355.00.0	PC000774.00.0	NA	hypothetical protein
PF14_0250	181	1092	6,0		yes	yes		PB000864.01.0	PC300698.00.0	PY07137	hypothetical protein
PFL0085c	268	1544	5,8			yes		NA	NA	NA	hypothetical protein
MAL13P1.149	201	1117	5,6			yes		PB000841.01.0	PC000409.01.0	NA	hypothetical protein
PF10_0126	333	1827	5,5			yes		PB000639.03.0	PC000639.00.0	PY01361	hypothetical protein conserved
PFA0200w	340	1854	5,5	yes		yes	yes	PB000881.01.0	PC000759.00.0	PY07092	S21 / TRSP (Kaiser et al 2004)
PF14_0582	689	3700	5,4			yes		NA	NA	PY05163	hypothetical protein
PFF0575c	175	922	5,3			yes		PB000579.01.0	NA	NA	hypothetical protein
PFE0360c	540	2798	5,2			yes		PB001289	PC001277.02.0	NA	hypothetical protein
PFI1230c	354	1801	5,1			yes		PB000780.01.0	PC000461.00.0	PY00930	hypothetical protein
PFL0915c	690	3465	5,0		yes	yes		PB000938.02.0	PC000139.00.0	NA	hypothetical protein
PF10_0283	3010	14670	4,9			yes		PB401461.00.0	PC000056.04.0	PY07110	hypothetical protein
PFL0445w	382	1852	4,8			yes		PB000558.02.0	PC000250.05.0	PY03793	hypothetical protein
MAL8P1.7	205	991	4,8			yes	yes	NA	NA	NA	hypothetical protein
PFE0365c	191	897	4,7			yes		NA	NA	NA	hypothetical protein
PFI0145w	827	3720	4,5			yes		PB000108.02.0	NA	PY01822	hypothetical protein
MAL13P1.268	232	1042	4,5		yes	yes		NA	NA	NA	hypothetical protein
PF10_0018	117	524	4,5		yes	yes		NA	NA	NA	alpha/beta hydrolase protein, putative
PF07_0067	486	2170	4,5			yes		PB000148.01.0	PC000489.04.0	PY01027	hypothetical protein
PFB0225c	2198	9794	4,5			yes	yes	NA	NA	NA	hypothetical protein
PFI0140w	4144	18333	4,4		yes	yes		NA	NA	NA	hypothetical protein
PFB0485c	222	935	4,2		yes	yes		NA	NA	NA	hypothetical protein
PF13_0116	283	1181	4,2		yes	yes		PB000489.00.0	PC000040.04.0	PY03873	hypothetical protein
PFI1675w	233	963	4,1			yes		NA	PC001412.02.0	PY05237	hypothetical protein
PFC0240c	187	740	4,0			yes		PB000797.03.0	PC000645.01.0	PY05028	hypothetical protein
MAL13P1.179	236	899	3,8			yes		PB105832.00.0	PC000837.02.0	PY01509	hypothetical protein
MAL13P1.141	129	476	3,7			yes		PB000243.03.0	PC000393.05.0	PY02849	hypothetical protein
PF13_0064	235	865	3,7			yes	yes	PB000846.02.0	PC301610.00.0	PY02806	hypothetical protein
PFI0670w	1007	3650	3,6			yes		PB001112.01.0	PC000704.04.0	PY02480	hypothetical protein conserved
MAL13P1.94	402	1449	3,6		yes	yes		PB301153.00.0	PC000868.01.0	NA	hypothetical protein
PF10_0257	235	834	3,5	yes		yes		PB107107.00.0	PC103405.00.0	PY03819	hypothetical protein
PF14_0535	1039	3579	3,4			yes	yes	PB000325.00.0	PC000732.03.0	PY00670	hypothetical protein
PFE1490c	286	985	3,4			yes		PB000665.01.0	PC000284.01.0	PY05158	RING zinc finger protein, putative
PFD0225w	558	1887	3,4		yes	yes		PB001071.02.0	PC000163.01.0	PY01338	hypothetical protein
PF08_0091	149	503	3,4		yes	yes		PB000198.02.0	PC001346.02.0	PY04682	hypothetical protein
PF11_0326	1300	4333	3,3			yes	yes	NA	NA	NA	hypothetical protein
PFL0840c	186	615	3,3			yes		PB001141.01.0	PC000137.02.0	PY05369	hypothetical protein
PF14_0732	402	1329	3,3			yes		NA	NA	NA	PHIST domain protein
PFA0440w	158	515	3,3			yes		PB000636.02.0	PC000179.05.0	PY05079	hypothetical protein
PFI1520w	205	664	3,2			yes		NA	NA	NA	asparagine-rich antigen, putative
PF14_0647	800	2535	3,2			yes		PB000826.01.0	PC000078.05.0	PY03684	hypothetical protein
PF10_0222	139	435	3,1			yes		NA	NA	NA	hypothetical protein
PF10_0112	1971	6117	3,1			yes		NA	PC000162.05.0	PY06766	hypothetical protein
PF11_0470	263	797	3,0		yes	yes		NA	NA	NA	hypothetical protein
PF10_0216	282	852	3,0		yes	yes		PB301571.00.0	PC301873.00.0	PY07622	hypothetical protein
PFD0115c	279	840	3,0			yes		NA	NA	NA	hypothetical protein
PFD1030c	184	555	3,0			yes		PB000870.03.0	PC000027.04.0	PY02465	hypothetical protein
PF14_0689	175	519	3,0			yes		PB101672.00.0	NA	NA	hypothetical protein
MAL8P1.25	659	1950	3,0			yes		NA	NA	PY01888	hypothetical protein
PF14_0019	190	562	3,0			yes		NA	NA	NA	hypothetical protein
PF14_0303	1210	3517	2,9			yes		PB000965.03.0	PC001381.02.0	PY04056	hypothetical protein
PFB0760w	360	1043	2,9		yes	yes		PB001130.02.0	PC000456.01.0	PY01249	MtN3-like protein, putative
PF14_0536	1357	3925	2,9			yes		PB000326.00.0	PC000000.04.0	PY02772	hypothetical protein

PFE1445c	218	629	2,9			yes		PB000995.02.0	PC302422.00.0	PY00695	hypothetical protein
PF14_0209	184	523	2,8			yes		PB000100.00.0	PC000184.01.0	PY07260	hypothetical protein
MAL8P1.80	797	2262	2,8			yes		PB000333.03.0	PC301050.00.0	PY00568	hypothetical protein
PFI0395w	256	723	2,8			yes	yes	PB001651.02.0	PC000042.03.0	PY06648	hypothetical protein
PFC0220w	306	851	2,8			yes		PB000855.02.0	PC000258.01.0	PY03898	hypothetical protein
PF10_0055	540	1502	2,8			yes		PB000604.03.0	PC000072.03.0	PY02980	hypothetical protein
PF10_0279	209	582	2,8			yes		PB000592.01.0	PC000235.03.0	PY05511	hypothetical protein
PFL1325c	301	832	2,8			yes		NA	NA	NA	hypothetical protein
PF08_0004	176	484	2,8		yes	yes		NA	NA	NA	hypothetical protein
PF14_0372	454	1248	2,7			yes	yes	PB000076.03.0	PC302506.00.0	PY00410	hypothetical protein /protein recongnized by volunteers immunized by irradied *P.falciparum* sporozoite (Doolan et al 2003)
PF10_0158	594	1631	2,7			yes	yes	PB000119.01.0	PC000920.01.0	PY01539	hypothetical protein
PF13_0137	929	2528	2,7			yes	yes	PB000665.00.0	PC001093.02.0	PY06638	hypothetical protein
PF14_0033	323	874	2,7			yes		PB104419.00.0	NA	PY06671	hypothetical protein
PFB0195c	375	1010	2,7			yes		PB300181.00.0	PC001340.02.0	PY00896	hypothetical protein
PFC0410w	265	715	2,7			yes		PB001648.02.0	PC000186.00.0	PY07308	YT521-B-like family protein, putative|conserved apicomplexan protein
PFI0800c	312	840	2,7			yes		PB000376.00.0	PC000393.03.0	NA	hypothetical protein
PF07_0101	285	764	2,7			yes		PB000085.00.0	PC000433.03.0	PY00052	hypothetical protein
PF10_0223	354	946	2,7	yes		yes		PB001058.02.0	PC300735.00.0	PY01666	hypothetical protein
PFA0215w	1875	4973	2,7			yes		PB000087.00.0	PC000812.00.0	NA	hypothetical protein
PF11_0067	266	700	2,6			yes		PB000362.00.0	PC000864.03.0	PY03777	hypothetical protein
PF14_0220	391	1022	2,6			yes		PB000460.02.0	PC300194.00.0	PY00020	hypothetical protein
PF14_0550	6467	16639	2,6			yes	yes	PB000933.01.0	PC301877.00.0	PY00674	hypothetical protein
PFL0045c	509	1309	2,6			yes		NA	NA	NA	PHIST domain protein
PFL1755w	453	1159	2,6			yes		PB001038.03.0	PC000680.04.0	NA	hypothetical protein
PF07_0116	164	419	2,6			yes	yes	PB000111.00.0	PC000812.04.0	PY00092	hypothetical protein
PFF0380w	186	474	2,5			yes		PB001068.03.0	PC001208.02.0	PY04854	hypothetical protein
PF10_0295	334	850	2,5		yes	yes		PB001030.02.0	PC000129.02.0	PY04569	hypothetical protein
PF14_0074	1064	2694	2,5			yes	yes	PB000372.00.0	PC000331.02.0	PY04135	hypothetical protein/ protein recongnized by volunteers immunized by irradied *P.falciparum* sporozoite (Doolan et al 2003)
MAL13P1.26	123	311	2,5			yes		PB000115.03.0	PC300225.00.0	PY02189	hypothetical protein
PFD0915w	325	820	2,5			yes		PB000175.03.0	PC000078.02.0	PY05601	hypothetical protein
PFB0425c	1026	2574	2,5			yes	yes	PB000582.00.0	PC000469.03.0	PY00323	S17 (Kaiser et al 2004)
MAL13P1.295	234	583	2,5			yes	yes	NA	NA	NA	hypothetical protein
PFD0375w	464	1155	2,5			yes		NA	NA	PY03396	hypothetical protein
PFF0765c	899	2213	2,5			yes		PB000400.02.0	PC000954.01.0	PY07542	hypothetical protein
PFE0330w	248	609	2,5			yes		PB000034.00.0	PC000540.04.0	PY06258	hypothetical protein
PFD0595w	227	553	2,4			yes		PB000153.01.0	PC000047.02.0	PY05323	hypothetical protein
PF08_0080	251	605	2,4			yes		PB000001.00.0	PC001083.02.0	PY00460	hypothetical protein
PFB0923c	260	627	2,4		yes	yes		NA	NA	NA	hypothetical protein
PFC0760c	400	953	2,4			yes	yes	PB301415.00.0	PC000574.04.0	PY05757	hypothetical protein
PFE0335w	259	613	2,4			yes		PB000033.00.0	PC000320.01.0	PY04352	hypothetical protein
MAL8P1.11	888	2085	2,3			yes		PB001018.03.0	PC000150.01.0	PY06243	hypothetical protein
PF08_0061	291	683	2,3			yes		NA	PC000240.00.0	NA	hypothetical protein
PFC0095c	368	855	2,3			yes		PB000276.02.0	NA	NA	hypothetical protein
PF13_0182	378	879	2,3		yes	yes	yes	PB000355.02.0	PC000262.00.0	PY06413	hypothetical protein
PF10_0054	516	1193	2,3			yes	yes	PB001161.01.0	PC300034.00.0	PY02979	hypothetical protein
MAL13P1.308	265	602	2,3			yes	yes	PB108442.00.0	PC104862.00.0	PY01759	hypothetical protein
PF14_0371	341	777	2,3			yes		PB000862.01.0	PC000248.00.0	NA	hypothetical protein
PFE1550w	174	395	2,3			yes		NA	NA	PY07379	intergenic sequence
PF14_0583	264	599	2,3			yes		PB000810.00.0	NA	PY05162	hypothetical protein
PF10_0106	302	684	2,3			yes	yes	NA	PC000095.00.0	PY02088	hypothetical protein
PFL0920c	323	729	2,3			yes		PB000937.02.0	PC300167.00.0	PY01802	hypothetical protein
PFL1045w	225	505	2,2			yes		PB000729.01.0	PC001213.02.0	PY03214	hypothetical protein
PF11_0435	325	722	2,2			yes	yes	PB001075.03.0	PC301003.00.0	PY05500	hypothetical protein / protein recongnized by volunteers immunized by irradied *P.falciparum* sporozoite (Doolan et al 2003)
PFB0285c	263	582	2,2			yes	yes	NA	PC000892.02.0	PY00070	hypothetical protein
PFA0205w	211	466	2,2			yes	yes	PB301531.00.0	PC000730.03.0	PY06306	S24 (Kaiser et al 2004)
PF11_0439	1804	3971	2,2			yes	yes	PB001052.02.0	PC000038.03.0	PY04078	hypothetical protein
PFL0130c	287	628	2,2			yes		PB000981.01.0	PC000349.03.0	PY01520	hypothetical protein conserved
PF10_0213	4917	10715	2,2			yes	yes	PB000951.00.0	PC000275.04.0	PY01999	10b antigen putative
MAL7P1.102	190	410	2,2		yes	yes	yes	NA	PC301254.00.0	PY02458	hypothetical protein
PF11_0291	2452	5280	2,2			yes		PB000356.02.0	PC000333.03.0	PY02741	hypothetical protein
PF14_0204	228	489	2,1			yes		PB000263.03.0	PC000465.02.0	NA	hypothetical protein
PFB0660w	181	389	2,1			yes		PB000192.00.0	NA	PY03305	hypothetical protein
PF11_0474	177	379	2,1			yes		NA	NA	NA	hypothetical protein conserved
PF14_0379	207	438	2,1			yes		PB001164.00.0	PC000376.03.0	PY04304	hypothetical protein
PF14_0052	507	1063	2,1			yes		PB000707.01.0	PC001420.02.0	PY03672	hypothetical protein conserved
PFL1685w	268	561	2,1			yes		PB001294.00.0	PC000017.02.0	PY00507	hypothetical protein conserved
PFL0790w	208	435	2,1		yes	yes		PB000129.00.0	PC301370.00.0	PY00178	hypothetical protein
PF11_0493	52425	109587	2,1	yes	yes	yes		NA	NA	NA	hypothetical protein
PF10_0364	234	490	2,1			yes		PB108401.00.0	PC300084.00.0	NA	hypothetical protein

PFF0645c	270	564	2,1			yes		PB000363.02.0	PC000005.04.0	PY01670	integral membrane protein
PF14_0402	164	341	2,1			yes	yes	PB000562.00.0	NA	NA	hypothetical protein
PF14_0045	150	313	2,1		yes	yes	yes	NA	NA	NA	hypothetical protein
PFD1135c	332	689	2,1			yes		NA	NA	NA	hypothetical protein
PFD1105w	3513	7278	2,1		yes	yes		NA	PC401501.00.0	NA	hypothetical protein
PF14_0362	700	1438	2,1			yes		NA	NA	NA	hypothetical protein
MAL13P1.316	173	356	2,1			yes		PB300652.00.0	PC300180.00.0	PY04589	hypothetical protein
PFF0795w	902	1840	2,0		yes	yes		PB001518.02.0	PC000214.04.0	NA	hypothetical protein
PFL0220c	1355	2754	2,0		yes	yes		PB000699.03.0	PC102801.00.0	NA	hypothetical protein
PF14_0018	297	603	2,0			yes		NA	NA	NA	PHIST domain protein
PFI1220w	6743	13558	2,0			yes		NA	NA	NA	hypothetical protein
PFE1120w	393	790	2,0			yes	yes	PB000920.00.0	PC000530.04.0	PY00040	hypothetical protein
PF14_0740	244	490	2,0			yes	yes	NA	NA	NA	hypothetical protein

GENES DOWN-REGULATED IN SPOROZOITES INCUBATED AT 37°C WITH HEPATOCYTES

gene.ID	salivary Gland sporozoite mean intensity	sporozoite incubated 1 hr at 37C° with hepatocytes mean intensity	Microarray ratio sporozoite 1 hr at 37C° with hepatocytes / salivary gland sporozoite	Found in Axenic liver stage (Wang et al 2004)	predicted signal peptide/anchor (SignalP)	Containing at least 1 predicted transmembrane domains (TMAP, TMPRED, TOPPRED2 and	detected in sporozoïte using mass spectrometry (Florens et at 2002)	Presence of orthologue in *P. berghei* (tarun et al 2008)	Presence of orthologue in *P. chabaudi* (tarun et al 2008)	Presence of orthologue in *P. yoelii* (tarun et al 2008)	description
**metabolic processes**											
PF10_0275	416	143	0,34			yes		PB000126.02.0	PC000339.04.0	PY02951	protoporphyrinogen oxidase putative
PF11_0395	1882	662	0,35			yes	yes	PB001219.00.0	PC301754.00.0	PY04459	guanylyl cyclase
PF14_0246	944	386	0,41			yes	yes	PB000844.03.0	PC000344.02.0	PY00206	phosphoenolpyruvate carboxylase putative
PF13_0187	912	394	0,43	yes		yes		PB000886.01.0	PC000401.03.0	PY01409	DNA helicase, putative|conserved Plasmodium protein, unknown function
PFD0085c	731	351	0,48	yes	yes	yes	yes	NA	NA	NA	long chain acyl-CoA synthase, ATP dependent, family 1, isoform 6
PFC0126c	990	469	0,47			yes		PB000592.00.0	PC300700.00.0	PY03962	PFMNL-1 mitoNEET-like iron-sulfur protein, putative
**nucleic metabolism**											
PFC0850c	685	241	0,35			yes		PB000924.01.0	PC000118.04.0	PY00970	endonuclease/exonuclease/phosphatase family protein, putative
PF10_0217	381	161	0,42					PB000229.02.0	PC000212.01.0	PY01659	pre-mRNA splicing factor putative
PFE0630c	747	329	0,44			yes		PB000705.03.0	PC000646.04.0	PY04605	orotate phosphoribosyltransferase putative
PFF1400w	805	372	0,46			yes	yes	PB000608.00.0	PC000105.04.0	PY04171	RAP protein, putative
MAL7P1.10	306	152	0,50		yes	yes		NA	NA	NA	centrin
**protein metabolism**											
PFE0830c	1007	234	0,23		yes	yes		PB000202.02.0	PC000384.03.0	PY03311	GTP-binding translation elongation factor tu family protein
PF14_0327	818	228	0,28				yes	PB000590.02.0	PC302079.00.0	PY02559	methionine aminopeptidase type II putative
PF13_0069	313	97	0,31			yes	yes	PB001061.02.0	PC000085.01.0	PY02337	translation initiation factor if-2 putative
MAL8P1.86	651	205	0,32		yes	yes		NA	PC000582.01.0	NA	Sel3 protein|conserved Plasmodium selenoprotein, unknown function
PF13_0228	1350	553	0,41	yes				PB000942.03.0	PC000692.04.0	PY06397	40S ribosomal subunit protein S6 putative
PFL1425w	1214	518	0,43			yes	yes	PB000346.03.0	PC000787.00.0	PY07337	t-complex protein 1 gamma subunit putative
PFD0990w	3342	1455	0,44				yes	NA	PC000317.01.0	PY06329	ribosome recycling factor, putative
PFI1575c	604	272	0,45					PB300259.00.0	PC301190.00.0	PY03620	peptide release factor putative
PFB0550w	447	212	0,48			yes		PB000780.02.0	PC000129.05.0	PY03558	peptide chain release factor subunit 1 putative
PF13_0126	648	310	0,48			yes		PB000539.02.0	PC000359.04.0	PY01060	translation initiation factor EIF-2B subunit related
**ubiquitin signalling pathway**											
PF08_0020	2279	1133	0,50					PB000364.01.0	PC000138.02.0	PY00518	Ubiquitination-mediated degradation component, putative
**protease**											
PFB0335c	419	171	0,41			yes		NA	NA	NA	cysteine protease, putative|serine repeat antigen 6 (SERA-6)
**signalling**											
PFA0335w	524	195	0,37					PB000519.01.0	NA	PY01029	P. falciparum GTP binding protein RAB5
PFE1010w	1263	522	0,41			yes		PB000146.00.0	PC301296.00.0	PY02192	protein phosphatase 2c putative
**transportation**											
PFL2065c	362	138	0,38					PB000813.00.0	PC000913.01.0	PY04101	mitochondrial import inner membrane translocase subunit putative
PFE0445c	712	297	0,42			yes		PB000031.03.0	PC000152.05.0	PY04299	SNAP protein (soluble N-ethylmaleimide-sensitive factor Attachment Protein), putative
**blood stage surface proteins**											
PF10_0345	1172	402	0,34		yes			NA	NA	NA	merozoite surface protein-3
PFL2625w	344	123	0,36		yes	yes		NA	NA	NA	rifin
PFA0740w	707	254	0,36			yes	yes	NA	NA	NA	rifin
PF10_0177	2215	838	0,38		yes	yes		PB001168.00.0	PC000273.03.0	PY02573	erythrocyte membrane-associated antigen
PFL1385c	881	335	0,38		yes			PB000668.01.0	PC000196.03.0	PY02883	Merozoite Surface Protein 9
PF14_0001	917	351	0,38					NA	NA	NA	erythrocyte membrane protein 1 (PfEMP1) truncated pseudogene
PFE1640w	704	306	0,43					NA	NA	NA	erythrocyte membrane protein 1 (PfEMP1), truncated, pseudogene
PF13_0010	891	397	0,45			yes		NA	NA	NA	glycophorin binding protein family, Gbph

PFD0125c	326	155	0,48			yes		NA	NA	NA	stevor
PFA0010c	386	184	0,48			yes		NA	NA	NA	rifin
PFD1240w	882	434	0,49			yes		NA	NA	NA	rifin
**parasitophorous vacuole proteins**											
PF10_0019	1183	382	0,32		yes	yes		NA	NA	NA	early transcribed membrane protein 10.1, etramp 10.1
**hypothetical proteins**											
PFI1715w	1506	410	0,27					NA	NA	NA	hypothetical protein
PF13_0321	377	109	0,29					NA	NA	NA	hypothetical protein
PF14_0669	4167	1213	0,29					PB000087.02.0	PC000079.00.0	PY06654	hypothetical protein
PFF0640w	470	140	0,30		yes			NA	PC302065.00.0	NA	hypothetical protein
PF11_0421	1511	537	0,36					PB000080.02.0	PC000375.00.0	PY02872	hypothetical protein
PFL0600w	2416	901	0,37		yes			PB001041.00.0	PC001257.02.0	PY06936	hypothetical protein
PF13_0060	793	301	0,38					PB001137.00.0	PC301180.00.0	PY02801	hypothetical protein
PF10_0184	1082	435	0,40				yes	NA	NA	NA	hypothetical protein
PFC0500w	1026	414	0,40				yes	PB001146.02.0	PC000526.00.0	PY01712	hypothetical protein
PF11_0503	1210	491	0,41		yes		yes	NA	NA	NA	hypothetical protein
PFI1380c	1138	488	0,43					PB001581.02.0	PC000122.05.0	NA	hypothetical protein
MAL13P1.345	328	143	0,44					NA	NA	PY04218	hypothetical protein
MAL8P1.47	602	267	0,44					PB000536.01.0	PC301916.00.0	PY07232	hypothetical protein
MAL13P1.113	394	182	0,46					NA	NA	PY07140	conserved Plasmodium protein, unknown function
PFL1675c	752	351	0,47		yes		yes	PB001293.00.0	PC000267.04.0	PY03515	CPW-WPC family protein
PF10_0021	370	174	0,47		yes			NA	NA	NA	PHIST domain protein
MAL8P1.134	6532	3125	0,48					PB101950.00.0	PC106553.00.0	PY04695	ferlin like protein, putative
PF11_0387	4602	2188	0,48					NA	NA	NA	hypothetical protein
PF13_0169	2452	1200	0,49					PB001245.00.0	PC001215.02.0	PY02177	hypothetical protein
MAL8P1.94	3711	1840	0,50					NA	NA	NA	hypothetical protein
**hypothetical transmembrane proteins**											
MAL13P1.93	1589	420	0,26			yes		PB001086.02.0	PC000704.03.0	PY00187	hypothetical protein
PFL2115c	547	151	0,28		yes	yes	yes	PB000850.03.0	PC000842.01.0	PY01214	glucose inhibited division protein A homologue putative
PF11_0232	613	212	0,35			yes	yes	PB000262.00.0	PC301868.00.0	NA	hypothetical protein
MAL8P1.143	1785	633	0,35			yes		PB300828.00.0	PC000265.04.0	PY04434	hypothetical protein
PF07_0074	3568	1286	0,36			yes		PB000710.02.0	PC300736.00.0	PY03294	hypothetical protein
PFC0515c	410	153	0,38			yes		PB000290.00.0	PC001307.02.0	PY01708	TPR Domain containing protein
PF11_0146	1247	509	0,41			yes		PB000660.00.0	PC302215.00.0	PY00732	hypothetical protein
PF11_0484	2094	878	0,42			yes		PB000802.02.0	PC300068.00.0	PY02407	hypothetical protein
PF13_0219	2280	978	0,43			yes	yes	PB000649.03.0	PC000126.00.0	PY04556	hypothetical protein
PFF0075c	374	163	0,44			yes		NA	NA	NA	hypothetical protein
PFL1980c	604	267	0,44			yes		PB000554.01.0	PC300673.00.0	PY02222	hypothetical protein
PFI1780w	477	214	0,45			yes		NA	NA	NA	PHIST domain protein
PF14_0506	778	354	0,45			yes		PB000401.03.0	PC101841.00.0	PY00257	hypothetical protein
PF14_0705	485	222	0,46			yes		PB000511.00.0	PC000776.01.0	PY03693	hypothetical protein
PFD0170c	328	152	0,46			yes		PB001000.02.0	NA	PY02446	hypothetical protein
PFF0095c	525	253	0,48			yes	yes	PB000291.01.0	PC000376.00.0	PY02270	hypothetical protein
PFL1300c	314	152	0,49		yes	yes		PB300607.00.0	PC000382.05.0	PY04372	hypothetical protein
PF14_0249	1459	710	0,49		yes	yes	yes	PB000236.03.0	PC301442.00.0	PY03329	hypothetical protein
PF10_0185	600	293	0,49			yes		NA	PC001014.02.0	NA	hypothetical protein
PFB0615c	1335	656	0,49			yes		PB001287.00.0	PC300732.00.0	PY03936	hypothetical protein
