# Supplementary material for: Temperature Shift and Host Cell Contact Up-Regulate Sporozoite Expression of Plasmodium falciparum Genes Involved in Hepatocyte Infection
Source: PLoS Pathog. 2008 Aug 8;4(8):e1000121. doi: 10.1371/journal.ppat.1000121 (PMC2488394; doi:10.1371/journal.ppat.1000121)
Supplement: Table S2 — Primers used for Taqman RT-qPCR experiments. (0.01 MB PDF) [file ppat.1000121.s004.pdf]

Table S2. Primers used for Taqman RT-qPCR experiments

Gene	Real time PCR forward primer	Real time PCR reverse primer	Taqman® probe
P. falciparum 18S	CTTTTGAGAGGTTTTGTTACTTTGAGTAA	TATTCCATGCTGTAGTATTCAAACACAA	TCATAACAGACGGGTAGTCATGATTGAGTTC
MAL8P1.6	TTTATTTTCCCCTTGTACGTGTAAA	GCTCCTGATATAAGAGCAAATTTCTT	AGGATTATGGGAAGGAATGGGTTAAGGATAAA
PFL0065w/ LSAP-1	ACACTGCCTATTGAATATTTCGATT	GCACATACAGCTGATAAAATAATTAACTG	TGAAACCCTCAAAGAAAATGTCAAAATTCG
MAL8P1.17	TGGTACATTAAATGAAACCCCAATAA	GGAACCAGCCTTGACAAAGAA	TTTCGAATGGTCTGGATTCCCAACCAT
MAL7P1.150	CCAAATATCAGATTAAATGCCTTAATCC	GCCAAAACCAACAATTAAATGAGTT	TGGAGGTGGCCAAGAAAGAGGATTAAGATC
PFB0105c / LSAP-2	TGGCATTATAGTCATTCTCTTTTGA	CATTTCCACCATTTCTTCATTTT	TGAAAAGTTCGTTATGGAGTATTTGTGGGAA
PFA0245w	CGGCAGCATTTTTATTACCTTTA	ACCATTCCGCTTGCATTATT	TGAAACAACAAAACATACCAATGCTGAAGGT
MAL6P1.157	GATATGTGGACCTTGCCTATATCATTA	ATTCATGTTCGCAGGCAATATATAA	AGGGAGGAGCCAAAATTGATATATGGGATTA
PF07_0126	TGTAATGGGCAACTTATCTTCTGA	ATATAGTCCTCATATTGGGAAAATTGG	CTTTTCGCCATGTTGGATAAATGTAGCAATAAA
PFI0580c	GACCAATCCAGTACCTATAC CTAAAAA	CTGTTCTCTGGGCTTAGGAAAA	AAACAAGATGGAAGTCAAAATAACACCACAACA
PF14_0678	GATCAATGTACCATCGCTGTTTAC	GTTTCTGCATTCATTTAATATACCGTGTA	AACACATTAAATGCCGTAGAATCCAAACCA
PFE0355c	GATAAAGGATCTGCCGAATG	CTCTTAGTTTCAATGTATATGGAGATG	TCTCCTTCCGCATCTACATTTACTTCCAT
PFI0135c	GTTCATGGACAAAGTGGAGAAAC	GTAACAGCTCCTCTGTTCGAATC	ACCTTCACAACTTCGATCTACCGCTAATCA
PF08_0054	AAGGTACCACAAATCGAAGTTACATTC	CGTTGGTAATTGTAATATGGTTTTGTTTA	ATATCGATGCTAACGGTATCTTAAACGT
PF11_0344	CATACATTGCTACTACTGCTTTGTC	TGATTCTCTTTCGATTTCTTTCA	CCCATCGAAGTTGAAAACAATTTTCCA
PF14_0425	GATTAGCAGAAAGATGCAAAGAGTATTA	TGGCTGTGTCAATAACTAAAACTGT	AAGCTGGTGCAAGGTTTGCTAAATGGA
PF13_0201	GCATGTGCTGGACTTGCTTA	TGCAGGTTCTCCGGCATA	AAATTCGTAGTACCAGGAGCAGCAACACC
PFD0425w / SIAP-1	CCAAAATATTGCATAGGAAGCACAT	AAACGAGCAGAATATTTATCTGAAGAGA	AGACAAGTTTCCATTGAAGATATAGCAGGAAACC
PFD0430c	AAACGTAAGCACTTCATCTATTGAACA	CCAGCACTTCCAAAACCAA	TGGAGTTAGCGTAAAAGCAAAAATTCAAGCTC
PF08_0005 / SIAP-2	CTATTGCTACTGTTCATAATAACACAAGA	TTGACTCCTTCAAGTATTTCATATTCT	ATTTGGTTTAAGCCCCTCTGAAGTACCA
PFD0825c	ACCCTTTCTAAGTTCTCATGCCTAT	TGTTATCATCAGAGTGTTCAAAGCA	ACGAGCAACAAATTAACAGATTGAATGAGC
PFL0085c	TTGAGATATCCCCAAGGAAA	TTCTTCTCATCTGCACTATTCATT	AGGAGAAAATGTAATGGACGGAAATTTTG
PFC0210c	TCCCCAT GTAGTGTAACTTGTG	CACTGGAACATTTTTCCATTT	TTCAAGTTAGAATAAAGCCTGGCTCTGCT
PFL0800c	CAGAAGTAAAAAAACACGCAAAATC	TTGGTGGTTTAACATTTTCAGCTA	TCAAAGTAGGATTGCCATCATTCGAAAATC
