# Supplementary material for: Temperature Shift and Host Cell Contact Up-Regulate Sporozoite Expression of Plasmodium falciparum Genes Involved in Hepatocyte Infection
Source: PLoS Pathog. 2008 Aug 8;4(8):e1000121. doi: 10.1371/journal.ppat.1000121 (PMC2488394; doi:10.1371/journal.ppat.1000121)
Supplement: Table S3 — Influence of temperature storage of sporozoite on the pattern of a number of up-regulated genes. (0.08 MB PDF) [file ppat.1000121.s005.pdf]

Table S3. Influence of temperature storage of sporozoite on the pattern of a number of up-regulated genes.

Fold increase when compared to salivary gland sporozoite

≤2	≤4	≤6	≤10	≤14	≤20	≥20

		Sporozoites kept at 4°C after dissection prior incubation at 37°C with hepatocytes			Sporozoites kept at room temperature after dissection prior incubation at 37°C with hepatocytes		
		30 min	1hr	2hr	30 min	1hr	2hr
Continious up-regulation	MAL8P1.6	**6.7** ± 1.4	**13.8** ± 2.1	**41.5** ± 7.5	**3.1** ± 1.0	**11.0** ± 3.2	**45.6** ± 3.5
	PFL0065w	**8.5** ± 3.4	**10.1** ± 4.1	**48.8** ± 10.2	**2.5** ± 0.5	**15.6** ± 4.5	**28.7** ± 12.0
	PFI0580c	**4.4** ± 1.2	**3.7** ± 0.9	**6.3** ± 1.7	**9.2** ± 3.7	**9.7** ± 2.7	**11.6** ± 3.1
	PF08_0054	**2.1** ± 0.4	**3.9** ± 0.6	**15.6** ± 4.1	**2.2** ± 0.3	**3.5** ± 0.7	**20.3** ± 5.1
Transient up-regulation	PF11_0344	**1.5** ± 0.2	**2.3** ± 0.2	**1.3** ± 0	**0.9** ± 0.0	**3.2** ± 1	**1.5** ± 0.0
	PF14_0425	**3.0** ± 0.1	**2.5** ± 0.1	**1.7** ± 0.1	**1.9** ± 0.1	**2.7** ± 0.4	**1.7** ± 0.2
	PF13_0201	**1.7** ± 0.1	**2.2** ± 0.3	**0.9** ± 0	**1.7** ± 0.0	**2.7** ± 0.5	**1.8** ± 0.2
	PFD0425w	**2.7** ± 0	**1.2** ± 0	**0.8** ± 0	**2.0** ± 0.2	**2.6** ± 0.6	**2.0** ± 0.3
	PFD0430c	**2.0** ± 0.3	**2.6** ± 0.1	**1.8** ± 0.2	**2.7** ± 0.4	**2.2** ± 0.2	**1.1** ± 0.0
	PFD0825c	**2.9** ± 0.4	**2.0** ± 0.1	**1.8** ± 0.1	**2.5** ± 0.2	**3.5** ± 0.9	**1.9** ± 0.6
	PFL0085c	**1.4** ± 0.4	**2.0** ± 0.1	**1.5** ± 0.1	**2.1** ± 0.2	**2.9** ± 0.7	**2.6** ± 0.5

Expression levels of 13 genes in sporozoites incubated at 37°C in the absence or the presence of primary human hepatocytes for 30 min, 1 hr or 2 hr, were normalized to the expression levels measured for control salivary glands sporozoites stored a 4°C (left) or room temperature (right) (values assigned as 1). Up-regulation ratios are represented by a colour gradient.
